# Efficacy of a sonic toothbrush on plaque removal—A video-controlled explorative clinical trial

**DOI:** 10.1371/journal.pone.0261496

**Published:** 2021-12-22

**Authors:** Nadine Schlueter, Sarah Fiedler, Maxi Mueller, Clemens Walter, Julia C. Difloe-Geisert, Kirstin Vach, Carolina Ganss

**Affiliations:** 1 Department of Operative Dentistry and Periodontology, Division for Cariology, Center for Dental Medicine, Medical Center, Albert-Ludwigs-University, Freiburg, Germany; 2 Department for Restorative Dentistry and Periodontology, Dental Clinics, Dental School, University Medicine Greifswald, Greifswald, Germany; 3 Department of Periodontology, Endodontology and Cariology, University Center of Dental Medicine (UZB), University of Basel, Basel, Switzerland; 4 Institute of Medical Biometry and Statistics, Faculty of Medicine and Medical Center, University of Freiburg, Freiburg, Germany; 5 Department of Conservative and Preventive Dentistry, Dental Clinic of the Justus-Liebig-University Giessen, Giessen, Germany; Danube Private University, AUSTRIA

## Abstract

Clinical studies on the efficacy of sonic toothbrushes show inconsistent results, most studies have been conducted without sufficient supervision of appropriate toothbrush usage. Aims of the explorative clinical trial were therefore to investigate whether the usage of an activated sonic toothbrush reduces plaque more effectively than an inactivated one used as a manual toothbrush, and to which extent the correct use of such toothbrush plays a role in its efficacy. The clinical trial was designed as a video-controlled interventional study. Thirty participants (mean (±SD) age 22.9 (±2.5) years) were included, areas of interest were the buccal surfaces of the upper premolars and the first molar (partial mouth recording). Toothbrushing was performed without toothpaste in a single brushing exercise under four different conditions: switched off, habitually used as manual toothbrush, no instruction; switched on, habitually used as powered toothbrush, no instruction; switched off, used as manual toothbrush, instruction in the Modified Bass Technique; switched on, used as powered toothbrush, instruction in a specific technique for sonic toothbrushes. Brushing performance was controlled by videotaping, plaque was assessed at baseline (after 4 days without toothbrushing) using the Rustogi modified Navy-Plaque-Index and planimetry. Main study results were that plaque decreased distinctly after habitual brushing regardless of using the sonic brush in ON or OFF mode (p for all comparisons < 0.001). After instruction, participants were able to use the sonic brush in ON mode as intended, with only minor impact on efficacy. Using the toothbrush in OFF mode with the Modified Bass Technique was significantly less effective than all other conditions (p for all comparisons < 0.001). Under the conditions used, the sonic toothbrush was not more effective when switched on than when switched off, and there was no evidence that the correct use of the toothbrush was more effective than the habitual use.

## Introduction

Although many patients credibly assure that they spend a lot of time and effort on oral hygiene, plaque removal is often not sufficiently successful. Based on this clinical experience, powered toothbrushes were developed with the aim of taking over the complex movements necessary with the manual toothbrush and making plaque removal more effective. Meanwhile, powered toothbrushes are established oral hygiene aids, and a variety of products with different forms of toothbrush heads and types of motion are marketed. Modes of action are counter-oscillation, rotation-oscillation, circular, and sonic movements. The greatest evidence seems to exist for rotation-oscillation brushes showing a greater reduction of plaque and gingivitis index scores than manual brushes [1]. The overall effect size, however, is limited, thus the clinical relevance of this finding is still unclear.

Against this background, sonic toothbrushes seem to be promising as they are supposed to detach plaque microorganisms not only at the contact area of the bristles with the tooth surface but also through non-contact energy transfer [2]. In a review of in vitro studies investigating the efficacy of powered toothbrushes in non-contact biofilm removal all included studies consistently has shown that, although a huge variety of experimental settings were used, non-contact biofilm removal is possible under laboratory conditions and that the effect size of which ranges between 38% and 99% [2]. Studies on the clinical efficacy of sonic brushes, however, are inconclusive so far. In a review from the Cochrane Collaboration from 2014 [1], sonic powered toothbrushes reduced plaque (4 studies) and gingivitis scores (5 studies) better than manual toothbrushes in the short term, but not in the long term observation (1 study). No significant differences were found between sonic and manual brushes on plaque and gingivitis scores (7 and 9 studies, resp., for the short term period; 3 and 3 studies, resp., for the long term period). In contrast, another systematic review from 2017 [3] including 12 fully published studies and 8 abstracts demonstrated a significantly greater ability of high-frequency, high-amplitude sonic powered toothbrushes to reduce plaque and gingivitis scores compared to manual brushes. Compared to rotating-oscillating powered toothbrushes, however, sonic powered brushes were less effective [4, 5]. In contrast, a recent narrative review that included 12 studies concluded that, in the long term and with regard to gingival bleeding, sonic toothbrushes tended to be more effective than rotary and oscillating toothbrushes. However, it must be qualified that the number of studies is too small to draw a final conclusion [6].

All clinical studies included in the reviews mentioned above used plaque and gingivitis scores as parameters of interest, but in most of the trials it remains unclear whether the participants were instructed, and whether they used the toothbrushes correctly. This, however, is an important issue because video observation studies of habitual toothbrushing behavior have shown that many subjects act in an ineffective way. A common pattern is changing frequently between different areas of the dental arch, neglecting the oral tooth surfaces, and brushing with unorganized movements [7–9]. Most interestingly, subjects showed the same patterns of brushing behavior regardless of whether they used a powered or a manual toothbrush [10]. This means that toothbrushing behavior often follows ingrained patterns and is not adapted to the intended use of a toothbrush type.

Aim of the present video-controlled explorative clinical study was therefore to investigate whether the usage of a sonic powered toothbrush reduces plaque more effectively than manual brushing, and to which extent the correct use of such a toothbrush plays a role in its efficacy. The null hypotheses were that (I) the sonication of a toothbrush does not increase plaque reduction compared to the same brush used without sonication and that (II) a specific brushing technique has no impact on plaque reduction.

## Materials and methods

### Study design, setting, and participants

The study was a prospective and explorative, not-randomized, interventional study, in which periodontal healthy young adults were included. The study was approved by the local Ethics Committee of the University in Freiburg, Germany (No. 288/17). The study has been registered in the German Clinical Trials Register (DRKS00024431). It conformed to the Declaration of Helsinki and to the guidelines of Good Clinical Practice [11]. The report of the study is given according to the TREND statement [12].

**Fig 1:**
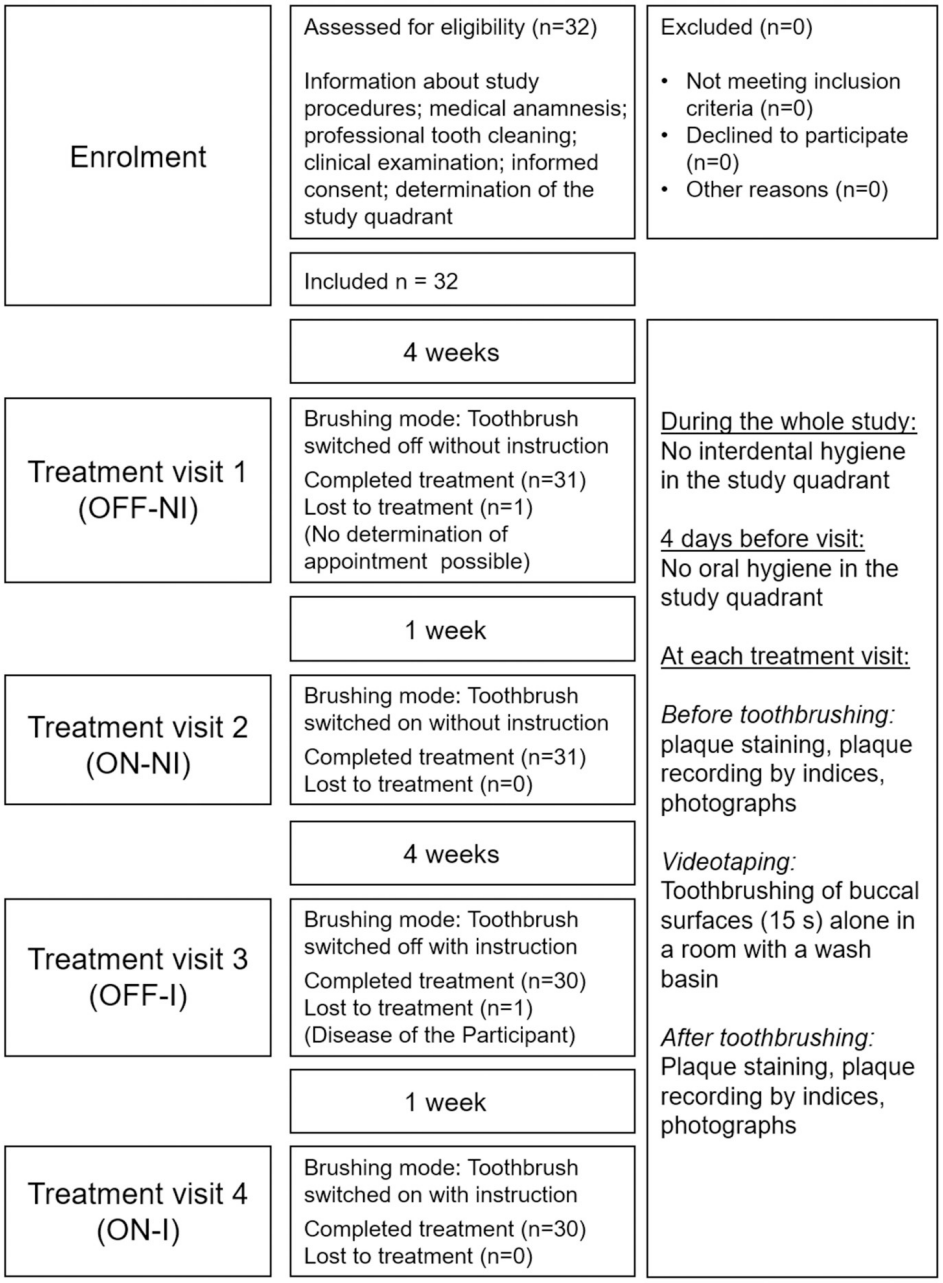
Flow chart of study procedures.

The clinical part of the study was conducted in the Dental Clinic, Department of Operative Dentistry and Periodontology, Center for Dental Medicine, University of Freiburg, which lasted from February to May 2019. One clinical investigator (S.F.) was responsible for recruitment, inclusion, clinical investigation, and instruction of participants as well as for videotaping and video analysis. One technical investigator (C.G.) was responsible for planimetric plaque analysis (for study flow chart see Fig 1).

Thirty-two participants were included in the study, from which, according to sample size calculation, 30 finished it (see Fig 1). The inclusion criteria were age of majority, informed consent, good general health (in particular no restrictions in manual abilities), fully dentate first or second quadrant (study quadrant = contralateral quadrant to the dominant hand) without proximal restorations, and probing depths > 4 mm or caries (diagnosed clinically visually and with near-infrared light trans-illumination images (DIAGNOcam; KaVo, Biberach, Germany)) in the premolar and molar region. Exclusion criteria were medication within the last 3 months that may have an influence on oral microorganisms (e.g. antibiotics, medications that may influence salivary flow), fixed orthodontic appliances, and regular use of any type of a powered toothbrush. All participants were dental or medical students in the preclinical section of studies at the University of Freiburg, Germany.

Eligible persons were informed about the study objectives and the study procedures. They received verbal and written information. In case of participation, volunteers gave written informed consent.

### Interventions

The study comprised five appointments for each participant (for details see flow chart in Fig 1). In the first appointment (enrolment), after inclusion, the study quadrant was individually determined as described above. All smooth surfaces of the teeth were professionally cleaned with a rubber cup (Prophy-Cup blue regular; Hager & Werken, Duisburg, Germany) and polishing paste (ProphyCare Prophy Paste CCS; RDA 120, Directa AB, Upplands Väsby, Sweden), the interdental spaces were cleaned with floss (Oral-B Essential Floss un-waxed; Procter & Gamble, Schwalbach am Taunus, Germany). Subjects were supplied with a standardized fluoridated toothpaste without additives having an impact on plaque formation (Dontodent Junior; dm-drogerie markt, Karlsruhe, Germany), and were informed that in the study quadrant, oral hygiene must not be performed for a total of four days before each appointment.

**Fig 2:**
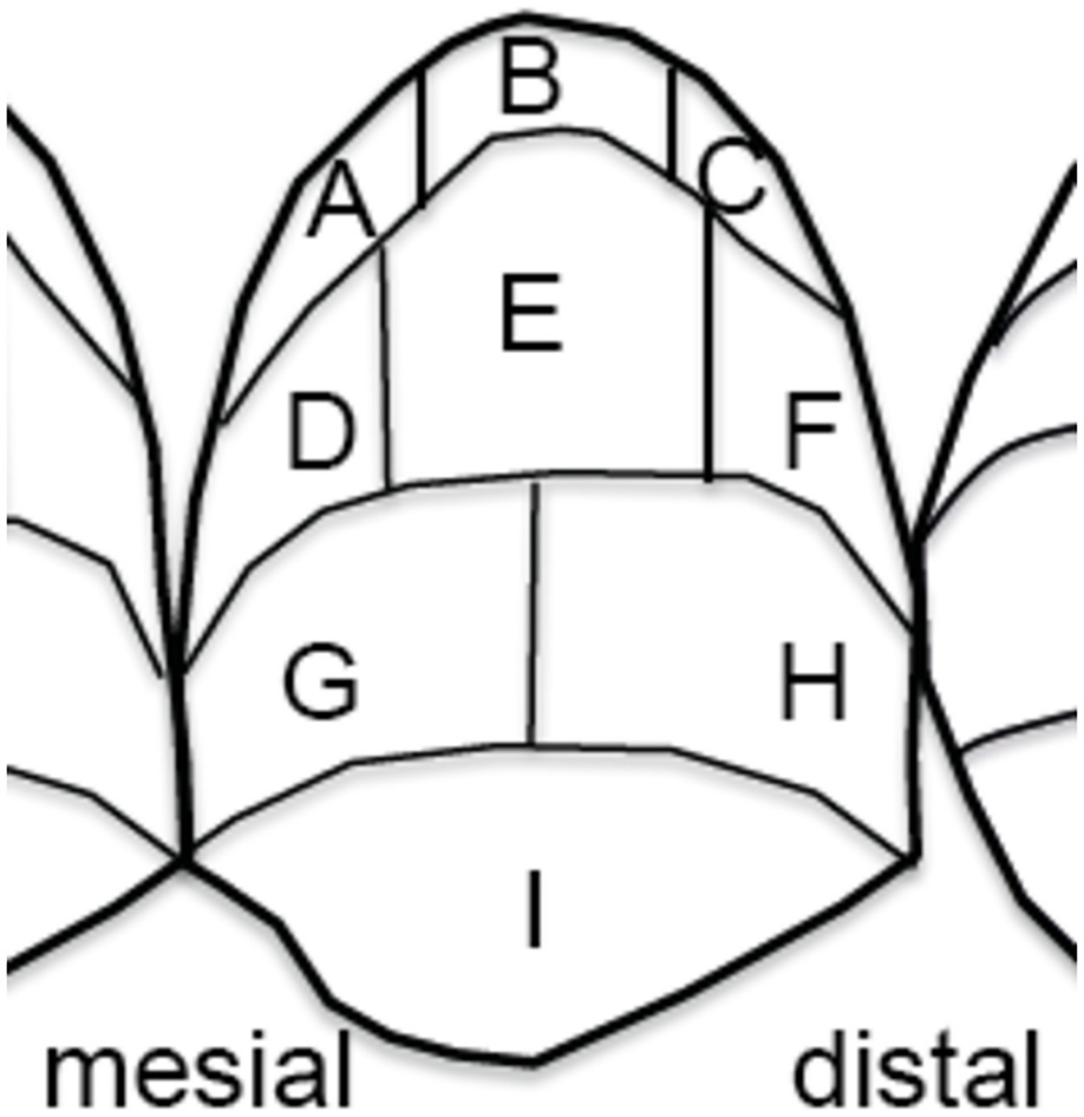
Areas of the Rustogi modified Navy-Plaque-Index (RMNPI). A and D represent the mesial proximal areas, C and F the distal proximal areas.

At the beginning of each appointment, (treatment visits 1–4), plaque was disclosed with a plaque revelator (Mira-2-Ton; Hager & Werken, Duisburg, Germany). The amount of plaque was scored with the Rustogi modified Navy-Plaque-Index (RMNPI, Fig 2) on the buccal surfaces of the first molar and the premolars in the study quadrant [13].

Afterwards, standardized photographs with a single lens reflex camera (Canon EOS 70D equipped with Canon EF 100mm f/2.8L Macro IS USM objective; Canon, Tokyo, Japan) were taken from the buccal surfaces of the study quadrant for planimetric quantification of plaque coverage. Then teeth were cleaned by the participants in the study quadrant (partial mouth recording, single brushing exercise) with a powered toothbrush (Sonicare ProtectiveClean 4300 (HX6848/92) equipped with a Premium Plaque Defence Standard brush head (HX9044/17); Philips, Hamburg, Germany) without toothpaste under four different conditions: (i) treatment visit 1, toothbrush switched off, habitually used as a manual toothbrush, no instruction (OFF-NI); (ii) treatment visit 2, toothbrush switched on, habitually used as a powered toothbrush, no instruction (ON-NI); (iii) treatment visit 3, toothbrush switched off, used as a manual toothbrush, instruction in the Modified Bass Technique (Table 1), (OFF-I); (iv) treatment visit 4, toothbrush switched on, used as a powered toothbrush, instruction in a specific technique for sonic toothbrushes (according to manufacturer’s instruction, Table 1), (ON-I). For each treatment visit, a new brush head was used.

**Table 1:** Description of instruction of the toothbrushing techniques.

Modified Bass Technique	Technique for the sonic toothbrush
placement of bristles under light pressure on the gingival margin at an angle of 45° to the longitudinal axis of the teeth, facing the root of the tooth bristles should slightly penetrate into the interdental spaces and into the sulcus small movements (half width of a premolar) of the brush head in horizontal direction while pressing against the tooth ten movements per tooth wiping out movement in occlusal direction continuing at the next tooth with the same procedure	placement of bristles without any pressure on the gingival margin at an angle of 45° to the longitudinal axis of the teeth facing the root of the tooth toothbrush should glide along the buccal surfaces without pressure in a slow forward and backward motion approximately 1.5 seconds per tooth continuing at the next tooth with the same procedure

Instructions in treatment visits 3 and 4 were given directly before brushing. Participants were videotaped during brushing procedure with a hidden camera (HC-VX 878; Panasonic, Kadoma, Japan) through a two-way mirror. Subjects were left alone during the brushing procedure and videotaping. Brushing was performed without toothpaste. After brushing, plaque was disclosed for a second time, the RMNPI was recorded, and photographs of the buccal surfaces were taken. In the last study appointment, all participants received a professional tooth cleaning.

### Objectives

The study aimed to answer the questions whether (I) a sonic toothbrush reduces plaque more effectively than the same brush used without sonication; (II) the dimension of plaque reduction depends on a specific brushing technique. For all brushing procedures, the same sonic toothbrush was either used with sonication switched off as a manual toothbrush (OFF mode) or with sonication switched on as a sonic toothbrush (ON mode).

### Measurement methods and outcomes

#### Toothbrushing behavior

Video analysis was performed with special software for observation of behavior (INTERACT 18; Mangold International, Arnstorf, Germany). Details of video analysis procedure are described elsewhere [14]. Videos were analyzed (S.F.) with regard to type of brushing movements (circling, horizontal, vertical, jiggling, jiggling-wiping (according to the Modified Bass Technique), passive (no specific technique), specific technique for sonic toothbrushes, and movement not assignable to the classes mentioned or not visible) in order to get information on the degree of adoption of instructed technique.

#### Planimetric plaque measurement

**Fig 3:**
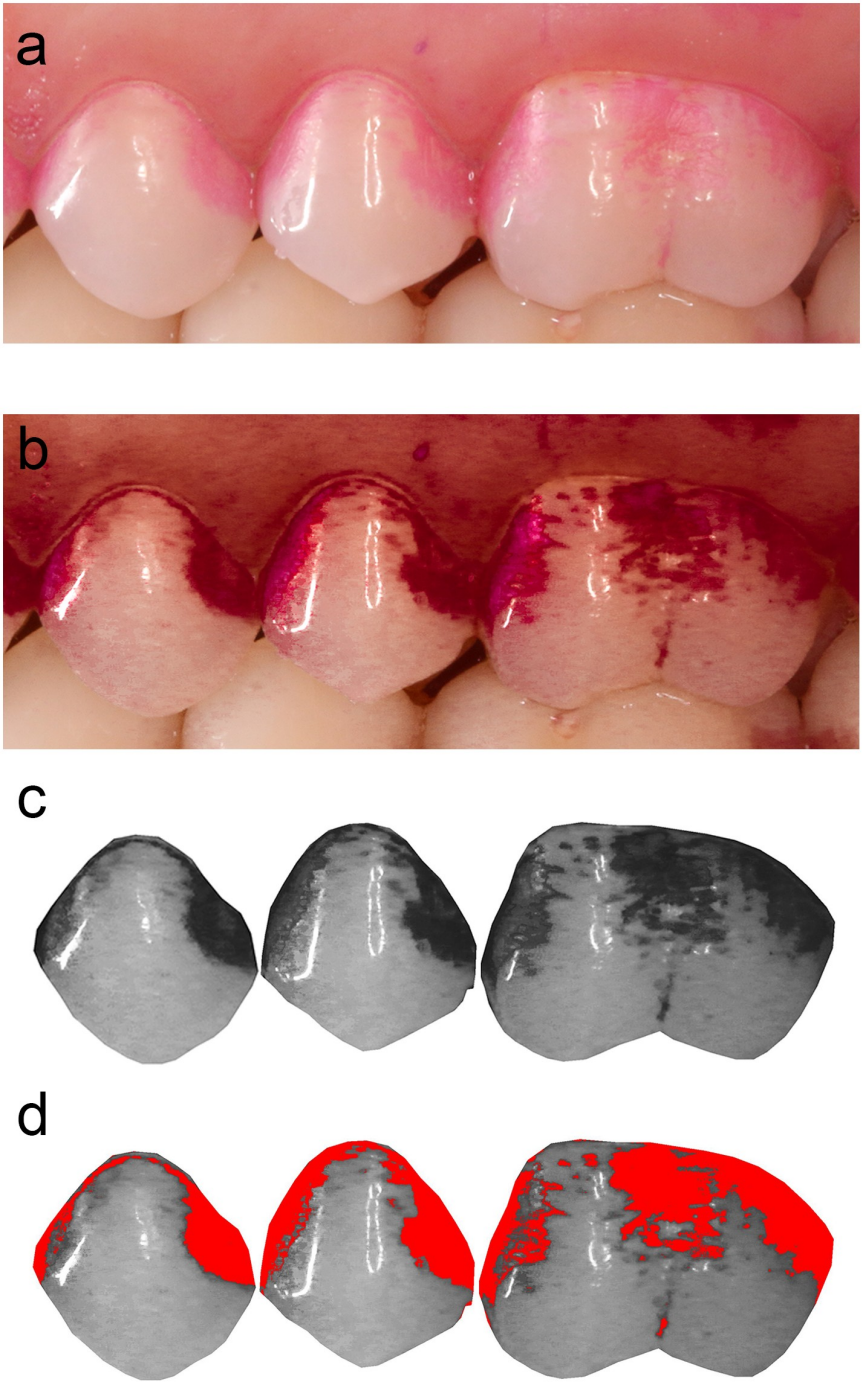
Principle of planimetric analysis. a) Original image; b) same image as (a), but after contrast enhancement; c) cropped tooth surfaces, converted into grey-scale; d) individually marked plaque covered areas, ready for automatic calculation of percentage of the tooth surface covered by disclosed plaque of the total tooth surface (P%D).

The processing of the images was blinded, performed by the technical investigator (C.G.) as shown in Fig 3, and was slightly modified as described earlier [15]. The original images (Fig 3a) were imported in an image processing program (Adobe Photoshop Lightroom Classic Version 8.3.1.; Adobe Systems, San Jose, California, USA). To enhance contrast, the saturation of red was set to +100 and the luminance to -100 (Fig 3b). Following, the images were further processed in another image processing program (ImageJ Version 1.47q; Wayne Rasband, National Institute of Mental Health, Bethesda, Maryland, USA). First, the region of interest was masked and the background cut, then the image was converted to 8 bit grey-scale (Fig 3c). The threshold value for defining a pixel as “plaque-covered” was set individually for each image (Fig 3d) in comparison to the original image, which was projected in parallel. The P%D (percentage of the tooth surface covered by disclosed plaque of the total tooth surface) was then automatically calculated.

#### Rustogi modified Navy-Plaque-Index

Plaque coverage was clinically measured with the Rustogi modified Navy-Plaque-Index (RMNPI, Fig 2). For this purpose, the smooth surface was divided into nine areas in each of which the occurrence of plaque was recorded dichotomously (plaque present, plaque absent). A sum score for areas of interest was calculated either for all areas (maximum of 9) or only for selected areas (mesial (AD) vs. distal (CF) proximal regions; gingival plus proximal areas (ABCDF) vs. the more central areas on the tooth surface (EGHI)).

### Sample size

The sample size calculation based on data from a study including a comparable study population [16]. After toothbrushing abstinence for about 12 hours, plaque values before (mean (±SD): 2.0 (±0.5)) and after brushing with a manual toothbrush were assessed (1.2 (±0.5)). Half of the reduction measured can be considered clinically relevant (plaque value reduction of 0.4). With the assumption that α = 0.05 and ß = 0.2, a sample size of 23 participants was calculated. Considering potential dropouts or withdrawals, a sample size of 30 participants was finally planned.

### Training, calibration, and reliability of analysis methods

All investigators were informed about all study procedures, and were thoroughly trained and calibrated. The clinical investigator (S.F.) was trained and supervised by an experienced clinician (M.M.), and calibrated with standardized photographs with regard to clinical plaque measurement as well as with standardized videos with regard to video analysis.

Intra- and interrater agreement of video analysis was calculated in INTERACT according to a previous study [9, 14]. Timed-event sequential data of multiple observers were compared based on kappa statistics. For all codes that recorded duration of type of brushing movements, overlapping = 85% and start tolerance = 0.96 s were set. For intra- and interrater agreement, 10 standardized videos from a previous study [14] were analyzed by S.F.. Intrarater agreement after training was κ = 0.861; interrater agreement was κ = 0.703.

The assessment of plaque values according to Rustogi modified Navy-Plaque-Index (RMNPI) was trained by repeated analysis of photographs of 25 teeth. Intrarater agreement was calculated by Cohens Kappa (κ = 0.916).

The reliability of the procedures for planimetric analysis, performed by C.G., were assessed by repeated analysis of the images (n = 24) of one randomly chosen subject. As these 24 images included pre- and post-brushing images, a wide range of P%D values was covered. The mean P%D (±SD) value was 28.6 (±31.9) for the first and 26.5 (±30.6) for the second analysis, the mean difference of which was 2.1 (±5.1). The intra-class correlation coefficient [95% confidence interval] was 0.985 [0.963; 0.994].

### Statistics

Results of analysis of brushing behavior are only given descriptively for estimation, whether participants adopted instructed brushing technique.

Other data were analyzed with statistics analyzing software (Stata Version 16.1; Stata, College Station, TX, USA). Level of significance was set to 0.05.

The planimetric data are expressed in percent (P%D; percentage of the tooth surface covered by disclosed plaque of the total tooth surface). Non-parametric tests were used for comparison between baseline and post-brushing P%D values (Wilcoxon matched-pairs signed-rank test). Impact of brushing condition on difference between baseline and post-brushing values measured with planimetry was analyzed with a linear mixed model with participant and tooth as random effects, and adjusting for the planimetric value at baseline. The method of Scheffe was used to correct for multiple testing in case of pairwise comparisons.

For analysis of RMNPI values per tooth, non-parametric tests were used for comparison between baseline and post-brushing (Wilcoxon matched-pairs signed-rank test). For comparison of differences between baseline and post-brushing values in specific areas within one brushing condition, also the Wilcoxon matched-pairs signed-rank test was used. Comparison of brushing conditions per area was performed using Δ%RMNPI values averaged over the three teeth examined. Analysis was performed with linear mixed model with the participant as random effect. Although the RMNPI values are discrete data, a regression analysis could be used because the distribution of the percentage difference between baseline and post-brushing values Δ%RMNPI met the requirements of a linear mixed model. The method of Scheffe was used to correct for multiple testing in case of pairwise comparisons. Additionally, Δ%P%D was calculated and presented descriptively to show the differences in discriminative power of the planimetry compared to the RMNPI.

## Results

Thirty-two participants were recruited, 30 of which finished the study (23 females, 7 males; dropout rate 6.3%). The mean age (±SD) of participants was 22.9 (±2.5) years (range 19–27 years). Twenty-nine participants were right-handed and one left-handed.

### Toothbrushing behavior

**Fig 4:**
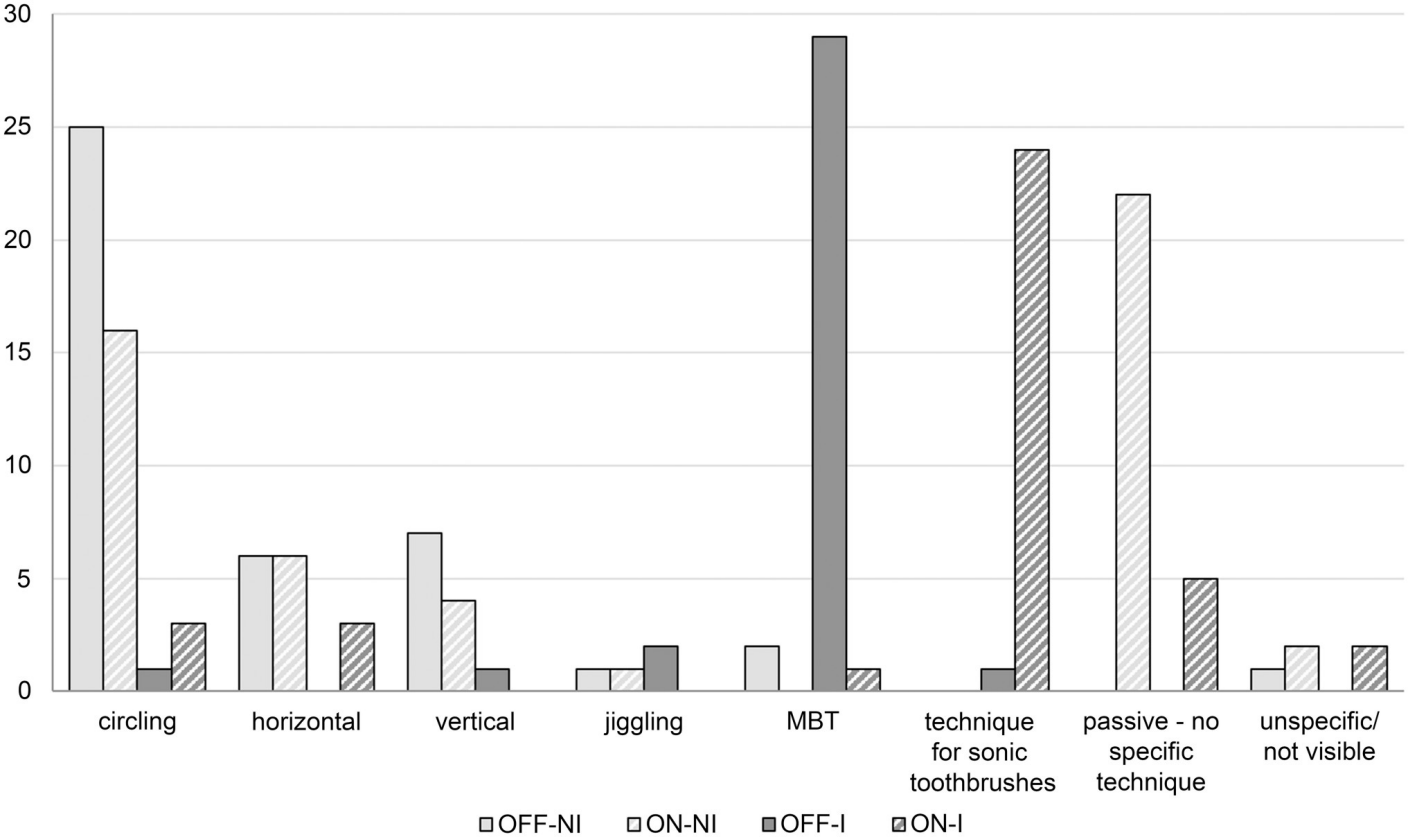
Occurrence of brushing movements on the buccal surfaces in the study quadrant. OFF-NI: habitual brushing using the toothbrush as a manual toothbrush; ON-NI: habitual brushing after activation of sonication; OFF-I: post instruction brushing using the toothbrush as a manual toothbrush; ON-I: post instruction brushing after activation of sonication; MBT: movements according to the Modified Bass Technique (combination of jiggling and wiping out); y-axis: number of participants showing a certain movement pattern; different types of movements can occur in one participant.

When using the toothbrush as a manual toothbrush without instructions (OFF-NI; habitual toothbrushing), participants mainly performed circling movements (Fig 4), followed by horizontal or vertical movement patterns. Instruction in the Modified Bass Technique (OFF-I) led to a distinct change in movement patterns. Nearly all participants used movements according to the Modified Bass Technique (combination of jiggling and wiping out) during the whole brushing time. When using the toothbrush without instructions with sonication switched on (ON-NI), the toothbrush was in most cases used passively without any specific technique directly followed by use of circling movements. Instruction into a special technique for sonic toothbrushes (ON-I) changed moving patterns and, in most cases, this specific technique was used.

### Planimetric plaque measurement

**Fig 5:**
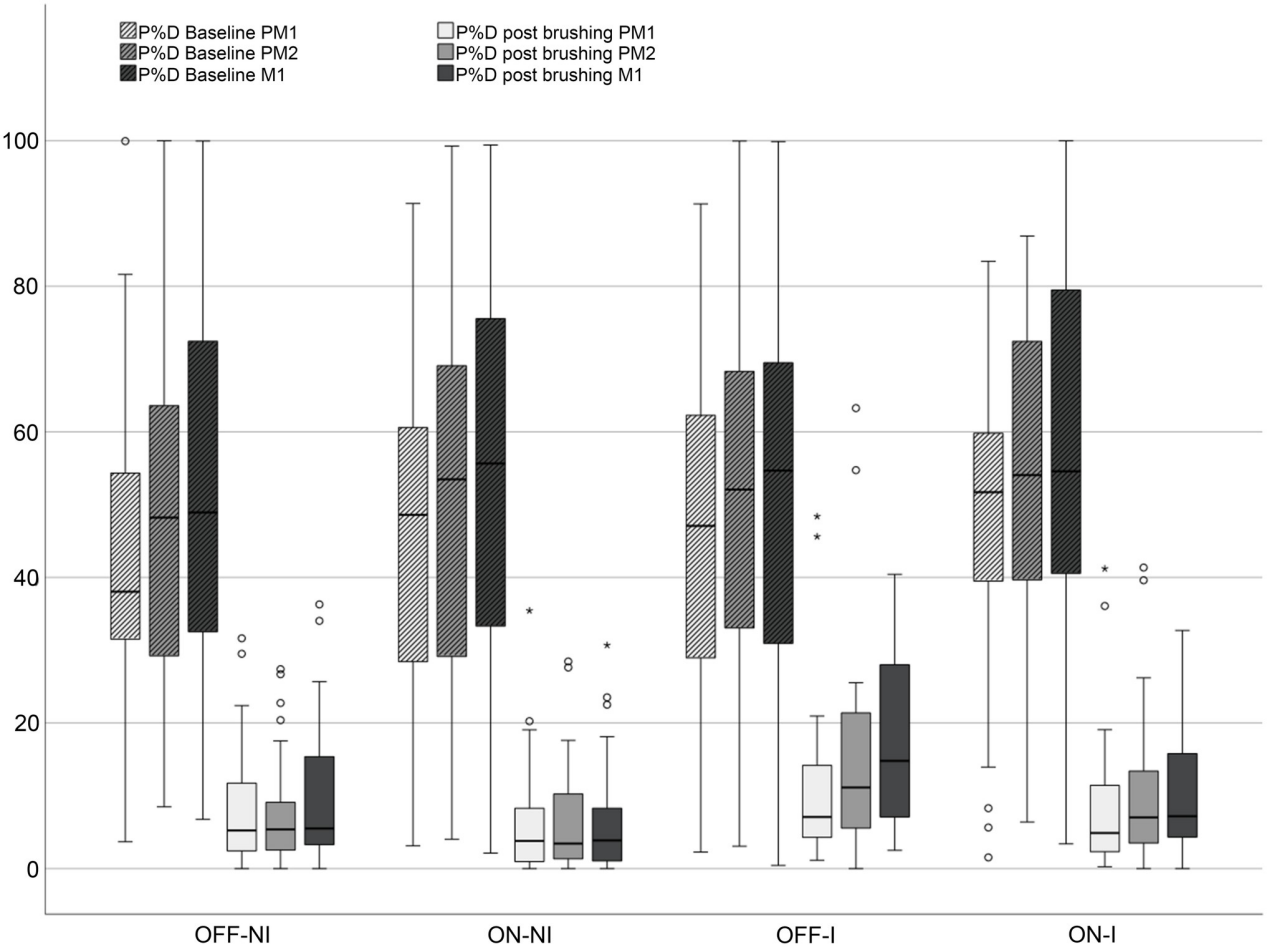
Boxplots of disclosed plaque of the total tooth surface (P%D) per tooth at baseline and post-brushing. All differences in P%D values between baseline and post-brushing were significant. Baseline: after 4 days refraining from oral hygiene; Post-brushing: after a single brushing exercise. OFF-NI: habitual brushing in OFF mode; ON-NI: habitual brushing in ON mode; OFF-I: post instruction brushing with the Modified Bass Technique in OFF mode; ON-I: post instruction brushing according to the manufacturer in ON mode; PM1 = first premolar; PM2 = second premolar; M1 = first molar.

The results of the planimetric measurements are shown in Fig 5. At baseline, no significant differences of P%D were found between groups; there was a tendency towards higher P%D values in more distal areas.

Regardless of the brushing mode, the single brushing exercise led to a distinct percentage reduction of P%D compared to baseline (Δ%P%D) ranging between 59% and 80% (Table 2).

**Table 2:** Descriptive presentation of the percentage reduction between baseline and post-brushing for planimetric values (Δ%P%D), and for RMNPI values (Δ%RMNPI) averaged over the three teeth given as mean (±SD).

	Δ%P%D all teeth	Δ%RMNPI all teeth
**OFF-NI**	76.3 (±22.5)	40.3 (±26.6)
**ON-NI**	80.0 (±25.6)	45.7 (±26.8)
**OFF-I**	59.4 (±48.2)	26.6 (±21.2)
**ON-I**	77.6 (±19.2)	39.0 (±23.9)

OFF-NI: habitual brushing in OFF mode; ON-NI: habitual brushing in ON mode; OFF-I: post instruction brushing with the Modified Bass Technique in OFF mode; ON-I: post instruction brushing according to the manufacturer in ON mode. Results of the regression analysis are given in the text.

The regression analysis revealed no significant difference for differences between baseline and post-brushing values between the OFF and ON modes with habitual brushing (ON-NI vs. OFF-NI; p = 0.224). Even after instruction, brushing with the sonication switched on did not result in better plaque reduction than habitual brushing in the OFF mode (OFF-NI vs. ON-I; p = 0.837). For some comparisons, the instruction even led to less effective plaque removal: brushing with the Modified Bass Technique revealed higher plaque amounts than habitual brushing both in OFF (OFF-I vs. OFF-NI; p < 0.001) and ON (OFF-I vs. ON-NI; p < 0.001) modes. Habitual brushing without instruction in ON mode was also more effective than after instruction (ON-NI vs. ON-I; p = 0.028). However, the instruction gave better results when using the sonic toothbrush in the ON mode than cleaning with the Modified Bass Technique in the OFF mode (OFF-I vs. ON-I; p < 0.001).

### Rustogi modified Navy-Plaque-Index

**Fig 6:**
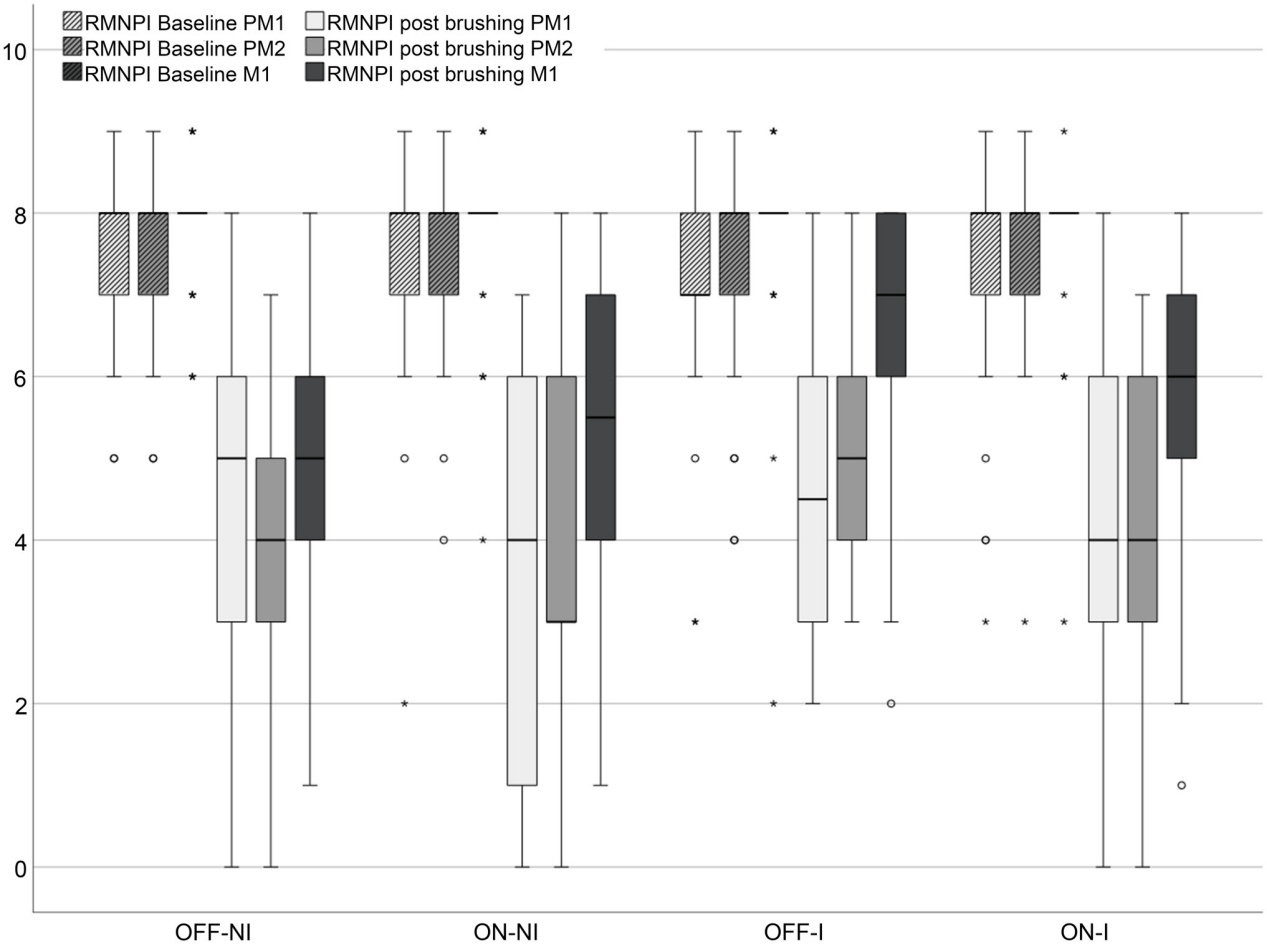
Boxplots of RMNPI values (range 0 to 9) per tooth at baseline and post-brushing. OFF-NI: habitual brushing in OFF mode; ON-NI: habitual brushing in ON mode; OFF-I: post instruction brushing with the Modified Bass Technique in OFF mode; ON-I: post instruction brushing according to the manufacturer in ON mode; PM1 = first premolar; PM2 = second premolar; M1 = first molar.

Rustogi modified Navy-Plaque-Index (RMNPI) values per tooth (Fig 6) did not differ between brushing conditions at baseline. Averaged over the three teeth examined, the RMNPI values were significantly decreased by brushing procedure regardless of brushing condition (p < 0.001) between 27% and 46% (Table 2). The lowest reduction was achieved by manual brushing after instruction (OFF-I), highest if the brush was used habitually with sonication switched on (ON-NI).

Independent of brushing condition, the reduction of plaque in the mesial proximal areas (AD) was significantly better than in the distal proximal areas (CF) (detailed values in Table 3; p < 0.01). The comparison of the plaque reduction between the gingival plus proximal areas (ABCDF) with the more central areas on the tooth surface (EGHI) revealed a significantly worse plaque reduction in the gingival plus proximal areas independent of brushing condition (p < 0.01).

**Table 3:** Percentage reduction (mean (±SD)) of RMNPI values (Δ%RMNPI) after brushing in selected areas.

Δ%RMNPI	AD	CF	ABCDF	EGHI
**OFF-NI**	32.8 (±31.4)^a^	15.2 (±19.0)^a^	33.5 (±20.1)^a^	53.2 (±28.1)^a^
**ON-NI**	44.2 (±32.4)^a^	20.6 (±24.0)^a^	40.2 (±22.4)^a^	56.9 (±29.8)^a^
**OFF-I**	20.6 (±23.4)^b^	4.0 (±10.6)^b^	20.6 (±13.2)^b^	39.5 (±26.4)^bc^
**ON-I**	34.3 (±30.3)^a^	15.0 (±21.6)^a^	34.1 (±19.3)^a^	49.6 (±27.6)^ac^

Different letters indicate statistical significance between brushing conditions within defined group of areas (within each column). OFF-NI: habitual brushing in OFF mode; ON-NI: habitual brushing in ON mode; OFF-I: post instruction brushing with the Modified Bass Technique in OFF mode; ON-I: post instruction brushing according to the manufacturer in ON mode; PM1 = first premolar; PM2 = second premolar; M1 = first molar.

## Discussion

The intention of the present study, to assess the impact of the sonic effect of a toothbrush as well as the application of a specific brushing technique on plaque removal, was investigated in a highly standardized study design including a video-controlling of the actual brushing procedure. This video control allowed us to verify that the intended use of the toothbrush was actually implemented, which makes our results particularly meaningful and shows the robustness of the study design.

The participants in our study were mainly dental or in a few cases medical students in their first semester. At the time of inclusion, the dental students had, according to the local regulations in effect for studies in dentistry, no contact with any dental course content. Therefore, they were not yet very knowledgeable about specific oral hygiene techniques, but they are already familiar enough with the subject to effectively change their toothbrushing behavior. This was evident in our video observations: before the instruction, the participants showed rather unspecific brushing and none used a specific brushing technique. After the instruction, almost all participants were able to perform the Modified Bass Technique correctly and almost all had adopted the specific technique for sonic toothbrushing.

The study area comprised the buccal surfaces of the maxillary premolars and first molar, as this is the area where most plaque growth occurs [15]. In order to create a uniform set of conditions for all subjects, professional cleaning of the teeth was performed on all subjects. This was performed with a polishing cup and paste for the smooth surfaces, and floss for the interdental spaces. Even though recent studies have shown that cleaning with an air-polishing system is more effective than using the conventional procedure with rubber cup and polishing paste [17], the cleaning as done in our study was deemed sufficient for the study question, particularly as it was performed identically for all subjects. The participants were not only asked to refrain from brushing their teeth before the examination appointment, but also not to perform interdental space hygiene before and during the entire study period. This was to ensure standardized plaque coverage as far as possible.

The amount of plaque was measured using two different methods. The RMNPI was used because it is one of the most complex plaque indices and is able to record plaque in a highly differentiated manner in the gingival and proximal areas. However, a disadvantage of this index is that it is not very discriminating. A tooth area that contains plaque is scored as positive without taking the amount or the dimension of plaque coverage in a respective area into account. Therefore, an additional planimetric evaluation was made. Planimetry is an objective method to quantify plaque, however, the proximal areas often cannot be adequately imaged due to the curvature of the teeth; uneven brightness of the image can make it difficult to determine the threshold value for pixels representing plaque. This problem was minimized by adjusting this threshold for each tooth individually until the setting matched the parallel projected clinical image as closely as possible. These two methods complement each other and we were thus able to analyze the effects of the different toothbrushing methods very comprehensively.

We only used a sonic toothbrush, but in different applications. If a test toothbrush is to be investigated, the control toothbrush is of great importance, since many influencing factors that could affect plaque removal have to be taken into account. Especially in the case of manual toothbrushes, there are many different shapes of brush heads and arrangements of bristles, the end rounding and hardness of which can also vary [18]. Especially the latter seems to be important for the effectiveness of plaque removal, because soft bristles may remove plaque to a lesser extent than medium-hard or hard bristles [19]. However, differences in plaque removal may also exist between different soft toothbrushes [20]. In order to avoid all these interferences, we have decided to use a sonic toothbrush when it is switched off, so that it can be used as a manual toothbrush. One can discuss whether the handle thickness has an impact on brushing performance, in particular if used as a manual toothbrush, which normally has a thinner handle. However, it can be assumed that this parameter plays a minor role in healthy individuals; the video analyses revealed a brushing behavior comparable to that observed in previous studies and, furthermore, it showed that the participants adopted the instructed technique.

The video analysis has shown that the brushing habits with the toothbrush used as a manual toothbrush (OFF mode) are consistent to findings of previous studies [9, 10], showing that young adults mostly brush with circling or horizontal scrubbing movements. Switching on the sonication (ON mode) resulted in a slightly more passive brushing procedure but with widely similar movements compared to the manual use. This also confirms the findings of a preceding study that investigated the brushing habits of subjects using oscillating-rotating powered toothbrushes [10]. Apparently, powered brushes, regardless of type, are often used like a manual toothbrush and not with the recommended more passive movements. Video analysis further revealed that after instruction, techniques were adopted by most participants. Even if such single instructions mostly have no lasting effects and training periods, remotivations and further instruction strategies are necessary to sustainably anchor new behaviors [21, 22], the dimension of this short-term adoption was more than sufficient for investigating the question under research.

As intended, the participants presented initially large amounts of plaque on the test teeth and there were no significant differences between baseline plaque levels at the four different time points. It is noteworthy that the range of initial plaque coverage between the thirty participants after four days of abstaining from oral hygiene was quite wide among the thirty participants, varying from about 10 to 100%, even if the teeth were all professionally cleaned at the beginning of the study (enrolment, Fig 1). This reflects the well-known fact that the plaque formation rate shows a high variability between healthy individuals [23] depending on individual composition of oral microbiota, sugar consumption, saliva parameters etc. or even on the type of cleaning procedure at baseline [17]. To cope with this problem in the regression analysis, data were adjusted for the planimetric value at baseline in agreement with the EMA guidance ‘Guideline on adjustment for baseline covariates in clinical trials’[24].

When the sonic toothbrush was used habitually, without prior instruction, the amount of plaque was significantly reduced regardless of the mode of use, ON mode or OFF mode. This was shown with the RMNPI as well as with the planimetric measurements. This means that at least when the sonic toothbrush is not used as intended, the sound effect seems to contribute little to the effectiveness of the toothbrush and that the first null hypothesis cannot be rejected for habitual brushing. But even after instruction and in ON mode (ON-I), plaque removal did not improve compared to habitual use in ON mode (ON-NI), meaning that the second null hypothesis also cannot be rejected for the use of the toothbrush in ON mode. However, looking at the two instruction groups, the sonic toothbrush was significantly more effective when used correctly and in ON mode (ON-I) than when the brush was used as a manual toothbrush in OFF mode with the Modified Bass Technique (OFF-I). The latter showed the worst results compared to all other groups. These results led also in parts to the rejection of the second null hypothesis “a specific brushing technique has no impact on plaque reduction” at least for the study design applied and the participants involved. This finding confirms a previous study that showed that the Modified Bass Technique leads to worse results than habitual brushing, even when the subjects had a practice period at home and fully adopted the technique.

In addition to the overall RMNPI areas, we looked at the more difficult-to-clean gingival and proximal areas individually, as sound activation could potentially offer some advantage here. However, the effects were the same as for the overall RMNPI index: plaque reduction was not influenced by the mode of application, except for the Modified Bass Technique, which was the least effective compared to all other groups.

A strength of our study is that the conditions of use of the sonic toothbrush in ON and OFF mode were well standardized and video-controlled. However, our study also has limitations. Even if we had controlled the intended use, the subjects only received a single instruction and implemented it directly afterwards. Further research would have to show whether the results could be improved with longer practice times and with some more habituation. Beyond this, a comparison to other powered toothbrushes might also be of interest. A recent narrative review has compared the efficacy of rotating-oscillating and sonic powered toothbrushes in plaque reduction and found that, due to the low number of studies, no superiority of a specific powered toothbrush over the other can be found. However, there was a tendency that in particular in the long-term use sonic powered toothbrushes are superior to other [6]. This clearly shows the necessity of further studies on this issue. In addition, we only examined a limited area of the dental arch, which shows the most plaque growth, but which is usually well reached during brushing. The effects in other regions of the dental arch, such as the oral surfaces, which are usually reached less well, need to be further investigated. Finally, the study investigated young adults, therefore our findings can only be transferred with caution to other groups of the population, e.g. patients with periodontal diseases or manual disabilities.

## Conclusions

The effectiveness of plaque removal was not improved by sound activation, even when the sonic toothbrush was used correctly according to the manufacturer’s instructions at least in the study population included and under the conditions used.

## Supporting information

S1 File (PDF)

S2 File (PDF)

S1 Data (XLSX)

S1 Checklist TREND statement checklist. (PDF)
