# Supplementary material for: Efficacy of a sonic toothbrush on plaque removal—A video-controlled explorative clinical trial
Source: PLoS One. 2021 Dec 22;16(12):e0261496. doi: 10.1371/journal.pone.0261496 (PMC8694435; doi:10.1371/journal.pone.0261496)
Supplement: S1 File — (PDF) [file pone.0261496.s001.pdf]

Plaque reduction as well as qualitative and quantitative composition of interdental biofilms after application of a sonic toothbrush

A clinical study

# - QIBS1 -

Protocol

September 2017

Prof. Dr. Nadine Schlüter
Division for Cariology, Department of Operative Dentistry and Periodontology, Center for Dental Medicine, Medical Center, Albert-Ludwigs-University, Freiburg, Germany

Prof. Dr. Carolina Ganß
Department of Conservative and Preventive Dentistry, Dental Clinic of the Justus-Liebig-University Giessen, Giessen, Germany

Prof. Dr. Clemens Walter
Department of Periodontology, Endodontology and Cariology, University Center of Dental Medicine (UZB), University of Basel, Basel, Switzerland

Table of Contents

1 Synopsis 3
2 Persons involved and responsibilities 5
2.1 Director of Studies 5
2.2 Other persons involved 5
2.3 Clinical investigator 6
2.4 Responsibilities 6
3 Scientific foundations 7
4 Aim of the planned investigation 7
5 Subjects, materials and methods 7
5.1 Subjects and recruitment 8
5.2 Criteria for drop-outs 8
5.3 Procedure 8
5.1. Flow Chart 11
5.4 Microbiological methods 11
5.5 Video observation and analysis 12
5.6 Planimetric determination of the plaque quantity 12
5.7 Randomisation and blinding 13
5.8 Review of the study by ethics committees 13
6 Plan for statistical analysis and case number calculation 13
7 Calibration and Training 14
8 Documentation 14
9 Quality assurance audits and controls 14
10 Amendments to the minutes 14
11 Publication 14
12 Literature 15

1 Synopsis

**TITLE OF TRIAL**	Plaque reduction as well as qualitative and quantitative composition of interdental biofilms after application of a sonic toothbrush - A clinical study
**SHORT TITLE**	Clinical effect of a sonic toothbrush
**EUDRACT NO**	Not relevant
**PROTOCOL NUMBER / INTERNAL PROTOCOL NUMBER**	QIBS1
**HEALTH CONDITION STUDIED**	Oral Health
**PHASE**	Not relevant
**OBJECTIVE(S)**	The aim of the study is the quantitative and qualitative analysis of interdental biofilms after application of a sonic toothbrush in ON mode compared to OFF mode. It is hypothesized that the extent, composition and structure of interdental biofilms differ when using the sonic toothbrush in ON mode compared to OFF mode.
**TREATMENT(S)**	1. clinical examination in the examination quadrant (tooth status, probing depths, exclusion of approximal caries by DiagnoCam); professional tooth cleaning of the entire dentition<br>2. Staining of plaque with a plaque revelator (erythrosine)<br>3. Toothbrushing instruction in the modified Bass technique<br>4. Toothbrushing instruction in the use of a sonic toothbrush
**INCLUSION CRITERIA**	Age of majority, Informed Consent, good general health (especially no physical restrictions), fully edentulous first or second quadrant (contralateral to the writing hand = examination quadrant) without proximal restorations, without probing depths >4 mm or caries in the premolar and molar region.
**EXCLUSION CRITERIA**	Within the last 3 months, taking medications that can influence oral microorganisms (e.g. antibiotics, medications that can influence salivary flow), orthodontic appliances, regular use of a sonic toothbrush.
**ENDPOINTS**	The study aims to answer the following questions:<br>• Is the extension of plaque on the tooth surface less after using the sonic toothbrush in ON mode than in OFF mode?<br>• Do the bacterial count and the composition of the biofilm in the interdental space differ after using the sonic toothbrush in ON mode compared to OFF mode?<br>• Are the effects from questions 1 and 2 dependent on a certain cleaning technique?
**TRIAL DESIGN**	The study is a single-blinded clinical observational study in healthy volunteers randomized to plaque sampling sites for evaluation of plaque coverage and microbiological analyses.

**STATISTICAL ANALYSIS**	Ratio scaled data (P%, total bacterial count, proportion of black pigmenting colonies, proportion of live or dead bacteria):<br>The data are checked for significant deviations from the normal distribution. Whether differences exist between the planimetrically determined plaque amount on the tooth surface (P%) and the microbiologically determined plaque amount in the interdental space after brushing in ON versus OFF mode under the different brushing techniques (bass technique in OFF mode versus habitual technique in OFF mode; bass technique in OFF mode versus correct technique in ON mode; habitual technique in OFF versus habitual technique in ON mode; instructed use in ON mode versus habitual use in ON mode) is investigated with t-tests for dependent samples.<br>If necessary, parameter-free procedures are used.<br>Ordinal scaled data (clinical plaque scores):<br>Whether differences exist between plaque scores after brushing in ON versus OFF mode under the different brushing techniques is investigated using the Wilcoxon test.	
**SAMPLE SIZE**	To be assessed for eligibility	n = 30
	To be allocated/randomised (if applicable) to trial	n = 30
	To be analysed	n = 30
**TRIAL DURATION**	Recruitment period (months):	About 3 months
	First patient in to last patient out (months):	About 5-6 months
	Treatment duration per patient (months):	10 weeks (within this time two oral hygiene instructions (in weeks 9 and 10)
	Follow up duration per patient (months):	10 weeks
**PLANNED DATES**	Enrolment of first patient, first patient in (FPI)	1st quarter 2018
	Enrolment of last patient, last patient in (LPI)	End of 2nd quarter 2018
	End of trial defined as last patient last visit (LPLV)	Beginning of Q3 2018
	Final statistical analysis	4th quarter 2018
	Planned interim analysis	None
**PARTICIPATING SITES**	Division for Cariology, Department of Operative Dentistry and Periodontology, Center for Dental Medicine, Medical Center, Albert-Ludwigs-University, Freiburg, Germany<br>Department of Conservative and Preventive Dentistry, Dental Clinic of the Justus-Liebig-University Giessen, Giessen, Germany<br>Department of Periodontology, Endodontology and Cariology, University Center of Dental Medicine (UZB), University of Basel, Basel, Switzerland	
**FUNDER(S)**	No external sponsoring planned, financing from the budget of the participating departments	

2 Persons involved and responsibilities

2.1 Director of Studies

Freiburg
Prof. Dr. Nadine Schlüter
Division for Cariology, Department of Operative Dentistry and Periodontology, Center for Dental Medicine, Medical Center, Albert-Ludwigs-University, Freiburg, Germany
Hugstetter Straße 55, 79106 D-Freiburg i. Br.
Email: nadine.schlueter@uniklinik-freiburg.de
Tel: 0049-761-270 48910, Fax: 0049-761-270 47390
Gießen
Prof. Dr. Carolina Ganß
Department of Conservative and Preventive Dentistry, Dental Clinic of the Justus-Liebig-University Giessen, Giessen, Germany
Schlangenzahl 14, 35392 D-Gießen
Email: carolina.ganss@dentist.med.uni-giessen.de
Tel: 0049-641-9946170, Fax: 0049-641-9946169
Basel
Prof. Dr. Clemens Walter
Department of Periodontology, Endodontology and Cariology, University Center of Dental Medicine (UZB), University of Basel, Basel, Switzerland
Hebelstrasse 3, CH-4056 Basel
Email: clemens.walter@unibas.ch
Tel: 0041-61-2672628, Fax: 0041-61-2672659

2.2 Other persons involved

Dr. Julia C. Schmidt
Department of Periodontology, Endodontology and Cariology, University Center of Dental Medicine (UZB), University of Basel, Basel, Switzerland
Hebelstrasse 3, CH-4056 Basel
Email: julia.schmidt@unibas.ch
Tel: 0041-61-2672623, Fax: 0041-61-2672659
Prof. Dr. Roland Weiger, Department of Periodontology, Endodontology and Cariology, University Center of Dental Medicine (UZB), University of Basel, Basel, Switzerland
Hebelstrasse 3, CH-4056 Basel
Email: roland.weiger@unibas.ch
Tel: 0041-61-2672618, Fax: 0041-61-2672659
Prof. Dr. Tuomas Waltimo,
Department of Periodontology, Endodontology and Cariology, University Center of Dental Medicine (UZB), University of Basel, Basel, Switzerland
Hebelstrasse 3, CH-4056 Basel
Email: tuomas.waltimo@unibas.ch
Tel: 0041-61-2672601, Fax: 0041-61-2672658
Dr. Eva Kulik Kunz
Department of Periodontology, Endodontology and Cariology, University Center of Dental Medicine (UZB), University of Basel, Basel, Switzerland
Hebelstrasse 3, CH-4056 Basel
Email: eva.kulik@unibas.ch
Tel: 0041-61-2672697, Fax: 0041-61-2672658

2.3 Clinical investigator

Doctorate student N.N. (Freiburg)

2.4 Responsibilities

Freiburg:

Prof. Dr. Schlüter is responsible for:
Study protocol, vote of the ethics committee, implementation and supervision of the clinical part of the study (recruitment of subjects, subject insurance, if necessary subject money, video observation and analysis, clinical plaque measurement, photos), publication.

Doctoral student N.N. is responsible for:
Conducting the clinical part of the study (inclusion of subjects, collection of clinical plaque indices, photographs, video observation and analysis, documentation and transmission of raw data).

Giessen:

Prof. Dr. Ganß is responsible for:
Study protocol, communication with local ethics committee, performance of planimetric plaque determination, publication.

Basel:

Prof. Dr. Walter is responsible for:
Study protocol and statistical design, conduct and supervision of the microbiological part of the study (microbiological analysis of plaque samples), statistical analysis, publication.

Dr. Julia C. Schmidt is responsible for:
Study protocol and statistical planning, implementation and supervision of the microbiological part of the study (microbiological analysis of plaque samples, documentation and transfer of raw data), statistical analysis, publication.

Dr. Eva Kulik Kunz is responsible for:
Study protocol and statistical planning, implementation and supervision of the microbiological part of the study (microbiological analysis of plaque samples, documentation and transfer of raw data), statistical analysis, publication.

Doctoral student N.N. is responsible for:
Conducting the microbiological part of the study (microbiological analysis of plaque samples, documentation and transfer of raw data).

3 Scientific background

In vitro studies show that the adhesion of bacteria to solid surfaces can be influenced by hydrodynamic effects. In particular, shear forces, surface tension forces and acoustic sound waves are capable of detaching adherent bacteria and eliminating biofilms in *vitro* [Sharma et al., 2005]. It is assumed that sonic toothbrushes can also produce hydrodynamic effects [Saxer et al., 2005]. This means that a cleaning effect is produced without direct bristle contact, if necessary even in less accessible areas.
A systematic review evaluated the efficacy of electric toothbrushes on biofilm removal without direct mechanical bristle contact [Schmidt et al., 2013]. The analysis of the studies included in this paper shows that various sonic toothbrushes can reduce biofilm without direct bristle contact in *vitro* (biofilm reduction in the range of 38 to 99%). A separate *in vitro* study analyzed the effectiveness of different sonic toothbrushes on biofilm removal without direct bristle contact [Schmidt et al., 2014]. The investigated sonic toothbrushes differed significantly with respect to their potential to remove a multispecies biofilm without direct bristle contact (biofilm reduction ranging from 9 to 80%). In another *in vitro* study, the hydrodynamic effect of four different sonic toothbrushes on proximal biofilm removal was investigated in an interdental model [Schmidt et al., 2016]. The sonic toothbrushes achieved biofilm reduction without direct bristle contact ranging from 7 to 64% and differed significantly in their effectiveness. The sonic toothbrushes that were able to achieve significant biofilm reduction operated at a higher frequency range (45,000 oscillations/min, 42,000 oscillations/min, 31,000 oscillations/min) than the toothbrush model that was unable to achieve significant biofilm reduction (26,000 oscillations/min). To date, there are no adequate clinical studies on a possible hydrodynamic effect of sonic toothbrushes.

4 Aim of the planned study

The aim of the study is the quantitative and qualitative analysis of interdental biofilms after application of a sonic toothbrush in ON mode compared to OFF mode. It is hypothesized that the extent, composition and structure of interdental biofilms differ when the sonic toothbrush is used in the ON mode compared to the OFF mode.

The study aims to answer the following questions:

- Is the extension of plaque on the tooth surface less after using the sonic toothbrush in ON mode than in OFF mode?
- Do the bacterial count and the composition of the biofilm in the interdental space differ after using the sonic toothbrush in ON mode compared to OFF mode?
- Are the effects from questions 1 and 2 dependent on a certain cleaning technique?

5 Subjects, materials and methods

The clinical parts of the study will be conducted at the Department of Operative Dentistry and Periodontology, Center for Dental Medicine, Medical Center, Albert-Ludwigs-University, Freiburg, Germany. The microbiological evaluation is performed at the Department of Periodontology, Endodontology and Cariology, University Center of Dental Medicine (UZB), University of Basel, Basel, Switzerland. Planimetric plaque determination is performed at the Department of Conservative and Preventive Dentistry, Dental Clinic of the Justus-Liebig-

University Giessen, Giessen, Germany.

The project follows the principles of Good Clinical Practice (ICH Harmonised Tripartite Guideline E6: Note for Guidance on Good Clinical Practice, CPMP/ICH/135/95 Step5) and the Declaration of Helsinki.

The study is a single-blinded clinical observational study in healthy volunteers randomized to plaque sampling sites for evaluation of plaque coverage and microbiological analyses.

5.1 Subjects and recruitment

The study group consists of 30 healthy volunteers (preclinical dental students) recruited by appropriate notices in the Center for Dental Medicine, Medical Center, Albert-Ludwigs-University, Freiburg, Germany.

Inclusion criteria: Age of majority, Informed Consent, good general health (especially no physical restrictions), fully edentulous first or second quadrant (contralateral to the writing hand = examination quadrant) without proximal restorations, probing depths >4 mm or caries in the premolar and molar region.

Exclusion criteria: taking medication within the last 3 months that may have an influence on oral microorganisms (e.g. antibiotics, medication that may influence salivary flow), orthodontic appliances, regular use of a sonic toothbrush.

5.2 Criteria for drop-outs

Discontinuation on the part of the subject, deviations from the protocol on the part of the subject (not completing all appointments, failure to carry out toothbrushing instructions), for health reasons: intermittent use of antibiotics or use of mouth rinses with an antibacterial effect.

5.3 Procedure

Appointment 1

The subjects are informed verbally and in writing about the objective, procedure and risks of the study, and finally the "informed consent" is obtained. The further inclusion and exclusion criteria are checked by means of a clinical examination in the examination quadrant (tooth status, probing depths, exclusion of proximal caries by DiagnoCam) and anamnesis questionnaire.

After inclusion, a professional cleaning of the entire dentition will be performed; especially in the examination quadrant, complete freedom from plaque should be achieved. Subjects are asked not to perform any interdental hygiene from the time of inclusion and for the duration of the study (at least for 4 weeks before the first examination appointment) and not to perform any oral hygiene in the examination quadrant for 4 days before the next appointment. The model of "de novo plaque accumulation" [Weiger et al., 1992] has proven effective in dental-clinical research for investigating the efficacy of different oral hygiene measures. When the subjects resume their usual daily oral hygiene, no negative consequences such as an increased risk of developing caries or permanent damage to the periodontium are to be expected. The test subjects use a standardized toothpaste without additives that may have an influence on oral microorganisms (Dontodent FluorFresh) during the entire trial period.

Appointment 2

Before and after brushing, plaque samples are taken from two different interdental spaces (mesial and distal to the second premolar of the examination quadrant, respectively); the order of collection is determined for each subject according to the randomization list. After taking the first plaque sample, plaque is stained with a plaque revelator (erythrosine) and the approximal plaque index [Lange et al., 1977] and the navy plaque index modified according to Rustogi et al. [Rustogi et al., 1992] are recorded on the buccal surfaces of the two premolars and the first molar, and a photograph is taken to determine the amount of plaque planimetrically.

Subsequently, under video observation, the subjects brush according to their home habits (habitual) with the sonic toothbrush (Philips® Sonicare FlexCare HX6902/02 Philips GmbH, Hamburg, Germany) in OFF mode. After brushing, a plaque sample is taken from the second interdental space and stained again, the two plaque indices are recorded and a photograph is taken. The subjects are again asked to refrain from any oral hygiene measures for 4 days before the next appointment in the examination quadrant.

Appointment 3

The 3rd appointment takes place 1 week after the 2nd appointment. The procedure corresponds to appointment 2, except that the subjects brush with the sonic toothbrush in ON mode. The subjects are again asked to refrain from any oral hygiene measures for 4 days before the next appointment in the examination quadrant.

Appointment 4

Four weeks after appointment 3, the next examination appointment takes place. The subjects receive toothbrushing instructions using the modified Bass technique. Subsequently, the procedure is the same as in appointment 2, but the subjects are now to use the toothbrush in OFF mode with the modified Bass technique. The subjects are again asked to refrain from any oral hygiene measures for 4 days before the next appointment in the examination quadrant.

Appointment 5

One week after appointment 4, the last examination appointment takes place. The subjects receive toothbrushing instruction in the use of a sonic toothbrush. Subsequently, the procedure is the same as in appointment 3, but the subjects are to use the toothbrush in ON mode according to the instructions for using a sonic toothbrush. Finally, all interdental spaces are professionally cleaned.

5.1. flow chart

Appointment 1

- Patient information ("Informed Consent")
- Medical history
- clinical examination (examination quadrant)
- Tooth cleaning of the entire dentition

↓ 4 weeks — no interdental hygiene

Appointment 2

- 4 days before: no oral hygiene measures
- Plaque sample, plaque staining, plaque indices, photo
- Tooth brushing under video-observation (habitual, OFF mode)
- Plaque sample, plaque staining, plaque indices, photo

↓ 1 week — no interdental hygiene

Appointment 3

- 4 days before: no oral hygiene measures
- Plaque sample, plaque staining, plaque indices, photo
- Tooth brushing under video-observation (habitual, ON mode)
- Plaque sample, plaque staining, plaque indices, photo

↓ 4 weeks — no interdental hygiene

Appointment 4

- 4 days before: no oral hygiene measures
- Tooth brushing instruction (modified bass technique)
- Plaque sample, plaque staining, plaque indices, photo
- Teeth brushing under video-observation (modified bass technique, OFF mode)
- Plaque sample, plaque staining, plaque indices, photo

↓ 1 week — no interdental hygiene

Appointment 5

- 4 days before: no oral hygiene measures
- Tooth brushing instruction (sonic toothbrush)
- Plaque sample, plaque staining, plaque indices, photo
- Tooth brushing under video-observation (cleaning technique, ON mode)
- Plaque sample, plaque staining, plaque indices, photo
- Professional tooth cleaning

5.4 Microbiological methods

The interdental space is dried with air and kept dry with the aid of suction and cotton rolls. The supragingival plaque is removed from the interdental space with sterile curettes and placed separately in a sterile tube filled with Reduced Transport Fluid (RTF) [Syed and Loesche, 1972]. The samples are pre-texted for one minute, glycerol is added to a final concentration of 20%, and then stored at -80°C. The samples are then removed from the tube.

The total bacterial count and the number of selected oral bacteria are determined by culture as well as by quantitative real-time PCR. To determine the total viable count (CFU/ml) and the percentage of black pigmenting colonies, the samples are pre-texted for one minute and appropriate dilutions are plated on blood agar plates (Columbia Agar Base [BBL Becton Dickinson, Allschwil, Switzerland] supplemented with 5mg/L hemin, 0.5 mg/L menadione and 50ml/L blood), incubated under aerobic and anaerobic conditions at 37 C° for 3-5 and 10 days, respectively, and then counted. For quantitative determination by real-time PCR, the genomic DNA of the bacteria is isolated from the samples and parts of the 16S rDNA are amplified. For this purpose, published primer pairs are used both for the quantitative determination of the total bacterial population (universal16S rDNA primer pair) and for the amplification of specific oral bacteria.

Live-Dead staining is used to determine the percentage of live or dead bacteria. Two specific fluorescent dyes (Live/Dead BacLight Bacterial Viability Kit; MoBiTec, Lucerne, Switzerland) are added to the samples. After appropriate incubation time, the samples are analyzed under a fluorescence microscope (CLSM; Carl Zeiss AG, Oberkochen, Germany). The staining is based on the permeability of the cell membrane. The dead cells fluoresce red, the living cells fluoresce green.

5.5 Video observation and analysis

The video observation takes place in the absence of the examiner over a period of 10 seconds. The subjects use the sonic toothbrush without toothpaste. The subjects brush in front of a mirror behind which a video camera is integrated. The procedure is described in detail elsewhere [Winterfeld, 2015]. Video analysis is performed after completion of the clinical part of the study using a special software for observing behavior (INTERACT® 15, 2015. Mangold International GmbH, Arnstorf, Germany). The procedure is described in detail elsewhere [Winterfeld, 2015].

The following parameters are collected:

- Brushing movement (circular, horizontal-linear, vertical-linear, vertical-rolling, shaking, shaking-wiping (modif. Bass), passive = brush head is only positioned on the tooth, no own brushing movements).

5.6 Planimetric determination of the plaque quantity

The buccal surfaces of the two premolars and the first molar are digitally photographed to fill the format. Stained plaque is quantified planimetrically by computer.

To do this, the color photos are first converted to grayscale (Adobe Photoshop Lightroom 5.4; Adobe Systems; San José, California, USA). On an 8-bit grayscale, 0 corresponds to pure black and 255 to pure white. The threshold for defining a pixel as "plaque-covered" is set at 90. Further processing (selection mask and automatic quantification) is performed using

ImageJ version 1.47q (Wayne Rasband; National Institute of Mental Health, Bethesda, Maryland, USA). The amount of plaque is described as the percentage of plaque-covered tooth surface to total tooth surface (P%). Target teeth are the two premolars and the first molar.

5.7 Randomization and blinding

Prior to the start of the study, two computer-generated randomization lists (plaque sample collection location before and after brushing; Microsoft Office Excel® 2011, Microsoft Corp., Redmond, WA, USA) and an appropriate list of subject codes will be generated. The intervention is identical for all subjects, so randomization is based only on the location of plaque collection.

The evaluation of the videos (Freiburg) is blinded with respect to the knowledge of the intervention; however, a recognition of the intervention due to the changed brushing habits cannot be completely excluded. The microbiological analyses (Basel) and the planimetric plaque determination (Gießen) are blinded to the intervention.

5.8 Review of the study by ethics committees

Prior to the start of the study, the project is first submitted to the Ethics Committee of the University of Freiburg; the project is then submitted to the Ethics Committees of the Universities of Basel and Gießen with a positive vote of the Ethics Committee Freiburg and, if necessary, their positive votes are awaited. The study will be registered in the clinical trials registry.

6 Plan for statistical analysis and case number calculation

Statistical analysis is performed with SPSS (SPSS® Statistics 23; SPSS Inc., Chicago, IL, USA). The statistical analysis is carried out in Basel.

Ratio scaled data (P%, total bacterial count, proportion of black pigmenting colonies, proportion of live or dead bacteria): The data are checked for significant deviations from the normal distribution. Whether differences exist between the planimetrically determined plaque amount on the tooth surface (P%) and the microbiologically determined plaque amount in the interdental space after brushing in ON versus OFF mode under the different brushing techniques (bass technique in OFF mode versus habitual technique in OFF mode; bass technique in OFF mode versus correct technique in ON mode; habitual technique in OFF versus habitual technique in ON mode; instructed use in ON mode versus habitual use in ON mode) is investigated with t- tests for dependent samples. If necessary, parameter-free procedures are used. Ordinal scaled data (clinical plaque scores): Whether differences exist between plaque scores after brushing in ON versus OFF mode under the different brushing techniques is investigated using the Wilcoxon test. The procedures for statistical analysis will be finalised after the completion of data collection.

Sample size calculation:
The sample size calculation is based on a study in which a comparable study population was included. In this study, plaque values were determined after toothbrushing abstinence for about 12 hours (omission of oral hygiene the evening before). A mean plaque value of 2.0±0.5 was measured. Subsequent oral hygiene with a manual toothbrush resulted in a plaque reduction to a mean plaque value of 1.2±0.5 [Van der Weijden et al., 2002].
Half of the reduction measured there can be considered clinically relevant (plaque value reduction of 0.4). Assuming $\alpha = 0.05$ and $ß = 0.2$ as well as a standard deviation of 0.5, this results in a group size of 23. Taking into account possible dropouts, a group size of 30 is therefore planned [Schlueter et al., 2010].

7 Calibration and training

The persons responsible for the study shall ensure that the persons involved in the clinical study as well as in the microbiological and plaque planimetric evaluations are informed in detail about the project and the objectives as well as the course of the study.
The clinical examiner (Freiburg) is calibrated with regard to the clinical plaque indices based on photos with stained plaque. In addition, the clinical examiner learns to evaluate these reproducibly according to the above-mentioned criteria using already existing videos.
Planimetric determination of plaque quantity (casting) is computer-automated by an experienced investigator, but data for reproducibility are also generated for this part of the study.

8 Documentation

All handwritten data are documented on forms (Case Report Form, CRF), which contain only the subject code. The entries are legible and complete. The film and photo files are only saved under the subject code. Backup copies of the files are made. The Informed Consent is the only document that allows the assignment of subject and subject code, and is kept separate from all other documents. The handwritten data and the data obtained with INTERACT are transferred to SPSS, checked for correctness (handwritten data) or for plausibility (INTERACT data) and evaluated. The archiving of the study documents including all raw data takes place in Freiburg.

9 Quality assurance audits and controls

The study directors at their centers ensure that the study is conducted according to protocol, guarantee the completeness of the documentation and the quality of the data. All centers allow themselves complete access to the study material and the subject data. The anonymity of the subjects is guaranteed and the data are handled confidentially.

10 Protocol amendments

Changes or deviations from the protocol are only permitted after discussion with all persons involved in the study. The responsible ethics committee(s) will be informed of protocol changes, the committee must agree if the changes are substantial (e.g. collection of additional data).

11 Publication

The results of the study should be published in a highly ranked journal. The results of the quantitative/qualitative microbiological plaque analysis of the clinical study will be published under first (position A) and last authorship of the group Basel with shared first authorship Freiburg/Gießen (position B), the results of the quantitative clinical plaque analysis of the clinical study will be published under first (position A) and last authorship of the group Freiburg/Gießen with shared first authorship (position B) Basel.
The results of the quantitative clinical plaque analysis of the clinical study (Freiburg) are also the subject of the dissertation of N.N.

12 Literature

Lange DE, Plagmann HC, Eenboom A, Promesberger A: Clinical methods for the objective evaluation of oral hygiene. Dtsch Zahnarztl Z 1977;32:44-47.

Rustogi KN, Curtis JP, Volpe AR, Kemp JH, McCool JJ, Korn LR: Refinement of the Modified Navy Plaque Index to increase plaque scoring efficiency in gumline and interproximal tooth areas. J Clin Dent 1992;3 (Suppl C):C9-C12.

Saxer UP, Imfeld T, van Waes H: Media release "Hydrodynamic sonic toothbrushes", SSO Taskforce 2010. Schweiz Monatsschr Zahnheilkd 2005.

Schlueter N, Klimek J, Saleschke G, Ganss C: Adoption of a toothbrushing technique: a controlled, randomised clinical trial. Clin Oral Invest 2010;14:99-106.

Schmidt JC, Astasov-Frauenhoffer M, Hauser-Gerspach I, Schmidt JP, Waltimo T, Weiger R, Walter C: Efficacy of various side-to-side toothbrushes for noncontact biofilm removal. Clin Oral Investig 2014;18:793-800.

Schmidt JC, Astasov-Frauenhoffer M, Waltimo T, Weiger R, Walther W: Efficacy of various side-to-side toothbrushes and impact of brushing parameters on noncontact biofilm removal in an interdental space model. Clin Oral Investig 2016;Oct 19 [Epub ahead of print].

Schmidt JC, Zaugg C, Weiger R, Walter C: Brushing without brushing?--a review of the efficacy of powered toothbrushes in noncontact biofilm removal. Clin Oral Investig 2013;17:687-709.

Sharma PK, Gibcus MJ, van der Mei HC, Busscher HJ: Influence of fluid shear and microbubbles on bacterial detachment from a surface. Appl Environ Microbiol 2005;71:3668-3673.

Syed SA, Loesche WJ: Survival of human dental plaque flora in various transport media. Appl Microbiol 1972;24:638-644.

Van der Weijden GA, Timmerman MF, Piscaer M, Snoek I, Van der Velden U, Galgut PN: Effectiveness of an electrically active brush in the removal of overnight plaque and treatment of gingivitis. J Clin Periodontol 2002 Aug 1;29:699-704.

Weiger R, Netuschil L, Brecx M: Relationship between bacterial counts, microbial vitality and the accumulation of supragingival dental plaque in humans. J Periodontal Res 1992;27:575-580.

Winterfeld, T. Toothbrushing and flossing habits in young adults: a video-based observational study. Med Diss, Giessen, 2015.
