# Supplementary material for: Efficacy of a sonic toothbrush on plaque removal—A video-controlled explorative clinical trial
Source: PLoS One. 2021 Dec 22;16(12):e0261496. doi: 10.1371/journal.pone.0261496 (PMC8694435; doi:10.1371/journal.pone.0261496)
Supplement: S2 File — (PDF) [file pone.0261496.s002.pdf]

Plaquereduktion sowie qualitative und quantitative Zusammensetzung interdentaler Biofilme nach Anwendung einer Schallzahnbürste

Eine klinische Studie

- QIBS1 -

Protokoll

September 2017

Prof. Dr. Nadine Schlüter
Stiftungsprofessur für Kariesforschung, Klinik für Zahnerhaltungskunde und Parodontologie, Department für Zahn-, Mund- und Kieferheilkunde, Universitätsklinikum Freiburg

Prof. Dr. Carolina Ganß
Poliklinik für Zahnerhaltungskunde und Präventive Zahnheilkunde, Justus-Liebig-Universität Gießen

Prof. Dr. Clemens Walter
Klinik für Parodontologie, Endodontologie und Kariologie, UZB-Universitätszahnkliniken, Basel

Inhaltsverzeichnis

1 Synopsis 3
2 Beteiligte Personen und Verantwortlichkeiten 5
2.1 Studienleiter 5
2.2 Weitere beteiligte Personen 5
2.3 Klinischer Untersucher 6
2.4 Verantwortlichkeiten 6
3 Wissenschaftliche Grundlagen 7
4 Ziel der geplanten Untersuchung 7
5 Probanden, Materialien und Methoden 8
5.1 Probanden und Rekrutierung 8
5.2 Kriterien für Drop-Outs 8
5.3 Ablauf 8
5.1. Flow Chart 11
5.4 Mikrobiologische Verfahren 12
5.5 Videobeobachtung und -analyse 12
5.6 Planimetrische Bestimmung der Plaquemenge 12
5.7 Randomisierung und Verblindung 13
5.8 Prüfung der Studie durch Ethik-Kommisionen 13
6 Plan zur statistischen Analyse und Fallzahlberechnung 13
7 Kalibrierung und Training 14
8 Dokumentation 14
9 Audits und Kontrollen zu Qualitätssicherung 15
10 Protokolländerungen 15
11 Publikation 15
12 Literatur 15

1 Synopsis

**TITLE OF TRIAL**	Plaquereduktion sowie qualitative und quantitative Zusammensetzung interdentaler Biofilme nach Anwendung einer Schallzahnbürste - Eine klinische Studie
**SHORT TITLE**	Klinischer Effekt einer Schallzahnbürste
**EUDRACT NO**	Nicht relevant
**PROTOCOL NUMBER / INTERNAL PROTOCOL NUMBER**	QIBS1
**HEALTH CONDITION STUDIED**	Mundgesundheit
**PHASE**	Nicht relevant
**OBJECTIVE(S)**	Das Ziel der Studie ist die quantitative und qualitative Analyse interdentaler Biofilme nach Anwendung einer Schallzahnbürste im ON-Modus im Vergleich zum OFF-Modus. Es wird angenommen, dass sich Ausdehnung, Zusammensetzung und Struktur interdentaler Biofilme bei Anwendung der Schallzahnbürste im ON-Modus im Vergleich zum OFF-Modus unterscheiden.
**TREATMENT(S)**	1. klinische Untersuchung im Untersuchungsquadranten (Zahnstatus, Sondierungstiefen, Ausschluss von Approximalkaries durch Diagnocam); professionelle Zahnreinigung des gesamten Gebisses<br>2. Anfärben von Plaque mit einem Plaquerevelator (Erythrosin)<br>3. Zahnputzinstruktion in der modifizierten Bass-Technik<br>4. Zahnputzinstruktion in der Anwendung einer Schallzahnbürste
**INCLUSION CRITERIA**	Volljährigkeit, Informed Consent, gute Allgemeingesundheit (insbesondere keine motorischen Einschränkungen), vollbezahnter erster oder zweiter Quadrant (kontralateral zur schreibenden Hand = Untersuchungsquadrant) ohne approximale Restaurationen, ohne Sondierungstiefen >4 mm oder Karies im Bereich der Prämolaren und Molaren.
**EXCLUSION CRITERIA**	innerhalb der letzten 3 Monate Einnahme von Medikamenten, die Einfluss auf orale Mikroorganismen haben können (z.B. Antibiotika, Medikamente, die den Speichelfluss beeinflussen können), kieferorthopädische Apparaturen, regelmäßige Verwendung einer Schallzahnbürste.
**ENDPOINTS**	Die Studie soll folgende Fragen beantworten:<br>• Ist die Ausdehnung von Plaque auf der Zahnoberfläche nach Anwendung der Schallzahnbürste im ON-Modus geringer als im OFF-Modus?<br>• Unterscheiden sich die Keimzahl und die Zusammensetzung des Biofilms im Interdentalraum nach Anwendung der Schallzahnbürste im ON-Modus im Vergleich zum OFF-Modus?<br>• Sind die Effekte aus den Fragen 1 und 2 abhängig von einer bestimmten Putztechnik?
**TRIAL DESIGN**	Die Studie ist eine auf die Entnahmestellen der Plaque bezogen randomisierte, auf die Auswertung der Plaquebedeckung und der mikrobiologischen Analysen bezogen einfach verblindete klinische Beobachtungsstudie an gesunden Probanden.

**STATISTICAL ANALYSIS**	Verhältnisskalierte Daten (P%, Gesamtkeimzahl, Anteil schwarzpigmentierender Kolonien, Anteil lebender bzw. toter Bakterien):<br>Die Daten werden auf signifikante Abweichungen von der Normal-Verteilung geprüft.<br>Ob zwischen der planimetrisch bestimmten Plaquemenge auf der Zahnoberfläche (P%) und der mikrobiologisch bestimmten Plaquemenge im Interdentalraum nach Putzen im ON- gegenüber dem OFF-Modus unter den verschiedenen Putztechniken (Bass-Technik im OFF-Modus versus habituelle Technik im OFF-Modus; Bass-Technik im OFF-Modus versus korrekte Technik im ON-Modus; Habituelle Technik im OFF- versus habituelle Technik im ON-Modus; Instruierte Anwendung im ON-Modus versus habituelle Anwendung im ON-Modus) Unterschiede bestehen, wird mit t-Tests für abhängige Stichproben untersucht.<br>Gegebenfalls kommen parameterfreie Verfahren zur Anwendung.<br>Ordinalskalierte Daten (klinische Plaquescores):<br>Ob zwischen den Plaquescores nach Putzen im ON gegenüber dem OFF Modus unter den verschiedenen Putztechniken Unterschiede bestehen, wird mit dem Wilcoxon-Test untersucht.	
**SAMPLE SIZE**	To be assessed for eligibility	n = 30
	To be allocated/randomised (if applicable) to trial	n = 30
	To be analysed	n = 30
**TRIAL DURATION**	Recruitment period (months):	Etwa 3 Monate
	First patient in to last patient out (months):	Etwa 5-6 Monate
	Treatment duration per patient (months):	10 Wochen (innerhalb dieser Zeit zwei Mundhygiene-instruktionen (in Wochen 9 und 10)
	Follow up duration per patient (months):	10 Wochen
**PLANNED DATES**	Enrolment of first patient, first patient in (FPI)	1. Quartal 2018
	Enrolment of last patient, last patient in (LPI)	Ende 2. Quartal 2018
	End of trial defined as last patient last visit (LPLV)	Anfang 3. Quartal 2018
	Final statistical analysis	4. Quartal 2018
	Planned interim analysis	Keine
**PARTICIPATING SITES**	Stiftungsprofessur für Kariesforschung, Klinik für Zahnerhaltungskunde und Parodontologie, Department für Zahn-, Mund- und Kieferheilkunde, Universitätsklinikum Freiburg<br>Poliklinik für Zahnerhaltungskunde und Präventive Zahnheilkunde, Justus-Liebig-Universität Gießen<br>Klinik für Parodontologie, Endodontologie und Kariologie, UZB-Universitätszahnkliniken, Basel	
**FUNDER(S)**	Kein externes Sponsoring geplant, Finanzierung aus dem Budget der beteiligten Abteilungen	

2 Beteiligte Personen und Verantwortlichkeiten

2.1 Studienleiter

Standort Freiburg

Prof. Dr. Nadine Schlüter
Stiftungsprofessur für Kariesforschung, Klinik für Zahnerhaltungskunde und Parodontologie, Department für Zahn-, Mund- und Kieferheilkunde, Universitätsklinikum Freiburg
Hugstetter Straße 55, 79106 D-Freiburg i. Br.
E-Mail: nadine.schlueter@uniklinik-freiburg.de
Tel: 0049-761-270 48910, Fax: 0049-761-270 47390

Standort Gießen

Prof. Dr. Carolina Ganß
Poliklinik für Zahnerhaltungskunde und Präventive Zahnheilkunde, Justus-Liebig-Universität
Schlangenzahl 14, 35392 D-Gießen
E-Mail: carolina.ganss@dentist.med.uni-giessen.de
Tel: 0049-641-9946170, Fax: 0049-641-9946169

Standort Basel

Prof. Dr. Clemens Walter
Klinik für Parodontologie, Endodontologie und Kariologie, UZB-Universitätszahnkliniken
Hebelstrasse 3, CH-4056 Basel
E-Mail: clemens.walter@unibas.ch
Tel: 0041-61-2672628, Fax: 0041-61-2672659

2.2 Weitere beteiligte Personen

Dr. Julia C. Schmidt
Klinik für Parodontologie, Endodontologie und Kariologie, UZB-Universitätszahnkliniken
Hebelstrasse 3, CH-4056 Basel
E-Mail: julia.schmidt@unibas.ch
Tel: 0041-61-2672623, Fax: 0041-61-2672659

Prof. Dr. Roland Weiger, Klinik für Parodontologie, Endodontologie und Kariologie, UZB-Universitätszahnkliniken, Hebelstrasse 3, CH-4056 Basel
E-Mail: roland.weiger@unibas.ch
Tel: 0041-61-2672618, Fax: 0041-61-2672659

Prof. Dr. Tuomas Waltimo,
Klinik für Präventivzahnmedizin und Orale Mikrobiologie, UZB-Universitätszahnkliniken, Hebelstrasse 3, CH-4056 Basel
E-Mail: tuomas.waltimo@unibas.ch
Tel: 0041-61-2672601, Fax: 0041-61-2672658

Dr. Eva Kulik Kunz
Klinik für Präventivzahnmedizin und Orale Mikrobiologie, UZB-Universitätszahnkliniken, Hebelstrasse 3, CH-4056 Basel
E-Mail: eva.kulik@unibas.ch
Tel: 0041-61-2672697, Fax: 0041-61-2672658

2.3 Klinischer Untersucher

Doktorand N.N. (Freiburg)

2.4 Verantwortlichkeiten

Freiburg:
Prof. Dr. Schlüter ist verantwortlich für:
Studienprotokoll, Votum Ethik-Kommission, Durchführung und Supervision des klinischen Teils der Studie (Rekrutierung von Probanden, Probandenversicherung, ggf. Probandengeld, Videobeobachtung und -analyse, klinische Plaquemessung, Fotos), Publikation.

Doktorand N.N. ist verantwortlich für:
Durchführung des klinischen Teils der Studie (Einschluss der Probanden, Erhebung der klinischen Plaqueindizes, Fotos, Videobeobachtung und -analyse, Dokumentation und Übertragung von Rohdaten).

Gießen:
Prof. Dr. Ganß ist verantwortlich für:
Studienprotokoll, Kommunikation mit der lokalen Ethik-Kommission, Durchführung der planimetrischen Plaquebestimmung, Publikation.

Basel:
Prof. Dr. Walter ist verantwortlich für:
Studienprotokoll und statistische Planung, Durchführung und Supervision des mikrobiologischen Teils der Studie (mikrobiologische Analyse der Plaqueproben), statistische Analyse, Publikation.

Dr. Julia C. Schmidt ist verantwortlich für:
Studienprotokoll und statistische Planung, Durchführung und Supervision des mikrobiologischen Teils der Studie (mikrobiologische Analyse der Plaqueproben, Dokumentation und Übertragung von Rohdaten), statistische Analyse, Publikation.

Dr. Eva Kulik Kunz ist verantwortlich für:
Studienprotokoll und statistische Planung, Durchführung und Supervision des mikrobiologischen Teils der Studie (mikrobiologische Analyse der Plaqueproben, Dokumentation und Übertragung von Rohdaten), statistische Analyse, Publikation.

Doktorand N.N. ist verantwortlich für:
Durchführung des mikrobiologischen Teils der Studie (mikrobiologische Analyse der Plaqueproben, Dokumentation und Übertragung von Rohdaten).

3 Wissenschaftliche Grundlagen

In vitro Studien zeigen, dass die Adhäsion von Bakterien an festen Oberflächen durch hydrodynamische Effekte beeinflussbar ist. Insbesondere Scherkräfte, Oberflächenspannungskräfte und akustische Schallwellen vermögen adhärierende Bakterien abzulösen und *in vitro* Biofilme zu eliminieren [Sharma et al., 2005]. [Es wird angenommen, dass Schallzahnbürsten ebenfalls hydrodynamische Effekte erzeugen können [Saxer et al., 2005]. Das bedeutet, eine Reinigungswirkung wird ohne direkten Borstenkontakt gegebenenfalls auch in weniger gut erreichbaren Arealen erzeugt.

Im Rahmen einer systematischen Übersichtsarbeit wurde die Wirksamkeit von elektrischen Zahnbürsten auf die Biofilmentfernung ohne direkten mechanischen Borstenkontakt evaluiert [Schmidt et al., 2013]. Die Analyse der in dieser Arbeit eingeschlossenen Studien zeigt, dass verschiedene Schallzahnbürsten einen Biofilm ohne direkten Borstenkontakt *in vitro* reduzieren können (Biofilmreduktion im Bereich von 38 bis 99%). In einer eigenen *in vitro* Studie wurde die Effektivität verschiedener Schallzahnbürsten auf die Biofilmentfernung ohne direkten Borstenkontakt analysiert [Schmidt et al., 2014]. Die untersuchten Schallzahnbürsten unterschieden sich signifikant hinsichtlich ihres Potenzials, einen Multispeziesbiofilm ohne direkten Borstenkontakt zu entfernen (Biofilmreduktion im Bereich von 9 bis 80%). In einer weiteren *in vitro* Studie wurde der hydrodynamische Effekt von vier verschiedenen Schallzahnbürsten auf die approximale Biofilmentfernung in einem Zahnzwischenraummodell untersucht [Schmidt et al., 2016]. Die Schallzahnbürsten erzielten eine Biofilmreduktion ohne direkten Borstenkontakt im Bereich von 7 bis 64% und unterschieden sich signifikant in ihrer Effektivität.. Die Schallzahnbürsten, die eine signifikante Biofilmreduktion erzielen konnten, arbeiten in einem höheren Frequenzbereich (45.000 Schwingungen/min, 42.000 Schwingungen/min, 31.000 Schwingungen/min) als das Zahnbürstenmodell, das keine signifikante Biofilmreduktion erreichen konnte (26.000 Schwingungen/min). Bislang liegen keine adäquaten klinischen Untersuchungen zu einem möglichen hydrodynamischen Effekt von Schallzahnbürsten vor.

4 Ziel der geplanten Untersuchung

Das Ziel der Studie ist die quantitative und qualitative Analyse interdentaler Biofilme nach Anwendung einer Schallzahnbürste im ON-Modus im Vergleich zum OFF-Modus. Es wird angenommen, dass sich Ausdehnung, Zusammensetzung und Struktur interdentaler Biofilme bei Anwendung der Schallzahnbürste im On-Modus im Vergleich zum Off-Modus unterscheiden.

Die Studie soll folgende Fragen beantworten:

- Ist die Ausdehnung von Plaque auf der Zahnoberfläche nach Anwendung der Schallzahnbürste im ON-Modus geringer als im OFF-Modus?
- Unterscheiden sich die Keimzahl und die Zusammensetzung des Biofilms im Interdentalraum nach Anwendung der Schallzahnbürste im ON-Modus im Vergleich zum OFF-Modus?
- Sind die Effekte aus den Fragen 1 und 2 abhängig von einer bestimmten Putztechnik?

5 Probanden, Materialien und Methoden

Die klinischen Teile der Studie werden in der Klinik für Zahnerhaltungskunde und Parodontologie, Department für Zahn-, Mund- und Kieferheilkunde des Universitätsklinikums Freiburg durchgeführt. Die mikrobiologische Auswertung erfolgt in der Klinik für Präventivzahnmedizin und Orale Mikrobiologie, UZB-Universitätszahnkliniken, Basel. Die planimetrische Plaquebestimmung erfolgt in der Poliklinik für Zahnerhaltungskunde und Präventive Zahnheilkunde der Justus-Liebig-Universität Gießen.

Das Vorhaben folgt den Grundsätzen der Guten Klinischen Praxis (ICH Harmonised Tripartite Guideline E6: Note for Guidance on Good Clinical Practice, CPMP/ICH/135/95 Step5) und der Deklaration von Helsinki.

Die Studie ist eine auf die Entnahmestellen der Plaque bezogen randomisierte, auf die Auswertung der Plaquebedeckung und der mikrobiologischen Analysen bezogen einfach verblindete klinische Beobachtungsstudie an gesunden Probanden.

5.1 Probanden und Rekrutierung

Die Studiengruppe umfasst 30 gesunde Probanden (Studierende der Zahnmedizin der Vorklinik), die durch geeignete Aushänge in der Klinik für Zahnerhaltungskunde und Parodontologie, Department für Zahn-, Mund- und Kieferheilkunde des Universitätsklinikums Freiburg rekrutiert werden.

Einschlusskriterien: Volljährigkeit, Informed Consent, gute Allgemeingesundheit (insbesondere keine motorischen Einschränkungen), vollbezahnter erster oder zweiter Quadrant (kontralateral zur schreibenden Hand = Untersuchungsquadrant) ohne approximale Restaurationen, Sondierungstiefen >4 mm oder Karies im Bereich der Prämolaren und Molaren.

Ausschlusskriterien: innerhalb der letzten 3 Monate Einnahme von Medikamenten, die Einfluss auf orale Mikroorganismen haben können (z.B. Antibiotika, Medikamente, die den Speichelfluss beeinflussen können), kieferorthopädische Apparaturen, regelmäßige Verwendung einer Schallzahnbürste.

5.2 Kriterien für Drop-Outs

Abbruch von Seiten des Probanden, Abweichungen vom Protokoll von Probandenseite (nicht alle Termine absolviert, fehlende Ausführung von Zahnputzinstruktionen), aus gesundheitlichen Gründen: intermittierende Antibiotikaeinnahme oder Anwendung von Mundspüllösungen mit antibakterieller Wirkung.

5.3 Ablauf

1. Termin

Die Probanden werden mündlich und schriftlich über Ziel, Ablauf und Risiken der Studie informiert, abschließend der „Informed Consent“ eingeholt. Die weiteren Ein-Ausschlusskriterien werden durch eine klinische Untersuchung im Untersuchungsquadranten

(Zahnstatus, Sondierungstiefen, Ausschluss von Approximalkaries durch Diagnocam) und Anamnesebogen geprüft.
Nach Einschluss wird eine professionelle Zahnreinigung des gesamten Gebisses durchgeführt, speziell im Untersuchungsquadrant sollte vollkommene Belagsfreiheit erreicht werden. Die Probanden werden gebeten, ab dem Einschlussdatum und für die Dauer der Studie (mindestens für 4 Wochen vor dem ersten Untersuchungstermin) generell keine Interdentalraumhygiene und 4 Tage vor dem nächsten Termin im Untersuchungsquandranten keinerlei Mundhygiene mehr durchzuführen. Das Modell der „de novo Plaque Akkumulation" [Weiger et al., 1992] hat sich in der zahnärztlich-klinischen Forschung zur Untersuchung der Wirksamkeit von unterschiedlichen Mundhygienemassnahmen bewährt. Bei Wiederaufnahme der gewohnten, täglichen Mundhygiene ist nicht mit negativen Folgen wie z.B. einem erhöhten Risiko für die Entstehung von Karies oder bleibenden Schäden am Zahnhalteapparat zu rechnen. Die Probanden verwenden während des gesamten Versuchszeitraums eine standardisierte Zahnpaste ohne Zusätze, die einen Einfluss auf orale Mikroorganismen haben können (Dontodent FluorFresh).

2. Termin

Vor und nach dem Zähneputzen werden Plaqueproben aus zwei verschiedenen Interdentalräumen (mesial bzw. distal des zweiten Prämolaren des Untersuchungsquadranten) entnommen, die Reihenfolge der Entnahme wird für jeden Probanden nach Randomisationsliste festgelegt. Nach Entnahme der ersten Plaqueprobe wird Plaque mit einem Plaquerevelator (Erythrosin) angefärbt und an den Bukkalflächen der beiden Prämolaren sowie dem ersten Molaren der Approximale Plaqueindex [Lange et al., 1977] und der nach Rustogi et al. modifizierte Navy Plaque Index [Rustogi et al., 1992] erhoben sowie ein Foto zur planimetrischen Bestimmung der Plaquemenge angefertigt.
Anschließend putzen die Probanden unter Videobeobachtung entsprechend ihren häuslichen Gewohnheiten (habituell) mit der Schallzahnbürste (Philips® Sonicare FlexCare HX6902/02 Philips GmbH, Hamburg, Deutschland) im OFF Modus. Nach dem Putzen wird eine Plaqueprobe aus dem zweiten Interdentalraum entnommen sowie nochmals angefärbt, die beiden Plaqueindizes erhoben und ein Foto angefertigt. Die Probanden werden erneut gebeten, vor dem nächsten Termin im Untersuchungsquadranten für 4 Tage auf jegliche Mundhygienemaßnahmen zu verzichten

3. Termin

Der 3. Termin findet 1 Woche nach dem 2. Termin statt. Der Ablauf entspricht Termin 2, nur putzen die Probanden mit der Schallzahnbürste im ON Modus. Die Probanden werden erneut gebeten, vor dem nächsten Termin im Untersuchungsquadranten für 4 Tage auf jegliche Mundhygienemaßnahmen zu verzichten

4. Termin

Vier Wochen nach Termin 3 findet der nächste Untersuchungstermin statt. Die Probanden erhalten eine Zahnputzinstruktion in der modifizierten Bass-Technik. Anschließend wird wie in Termin 2 verfahren, jedoch sollen die Probanden die Zahnbürste im OFF Modus nun mit der modifizierten Bass Technik verwenden. Die Probanden werden erneut gebeten, vor dem nächsten Termin im Untersuchungsquadranten für 4 Tage auf jegliche Mundhygienemaßnahmen zu verzichten.

5. Termin

Eine Woche nach Termin 4 findet der letzte Untersuchungstermin statt. Die Probanden erhalten eine Zahnputzinstruktion in der Anwendung einer Schallzahnbürste. Anschließend wird wie in Termin 3 verfahren, jedoch sollen die Probanden die Zahnbürste im ON Modus nach Anweisung zur Verwendung einer Schallzahnbürste verwenden. Abschließend werden alle Interdentalräume professionell gereinigt.

5.1. Flow Chart

1. Termin	Patienteninformation ("Informed Consent")<br>Anamnese<br>klinische Untersuchung (Untersuchungsquadrant)<br>Zahnreinigung des gesamten Gebisses	
	4 Wochen ↓	keine Interdentalraumhygiene
2. Termin	4 Tage vorher: keine Mundhygienemassnahmen<br>Plaqueprobe, Plaqueanfärbung, Plaqueindizes, Foto<br>Zähneputzen unter Vidobeobachtung<br>(habituell, OFF-Modus)<br>Plaqueprobe, Plaqueanfärbung, Plaqueindizes, Foto	
	1 Woche ↓	keine Interdentalraumhygiene
3. Termin	4 Tage vorher: keine Mundhygienemassnahmen<br>Plaqueprobe, Plaqueanfärbung, Plaqueindizes, Foto<br>Zähneputzen unter Vidobeobachtung<br>(habituell, ON-Modus)<br>Plaqueprobe, Plaqueanfärbung, Plaqueindizes, Foto	
	4 Wochen ↓	keine Interdentalraumhygiene
4. Termin	4 Tage vorher: keine Mundhygienemassnahmen<br>Zahnputzinstruktion (modifizierte Basstechnik)<br>Plaqueprobe, Plaqueanfärbung, Plaqueindizes, Foto<br>Zähneputzen unter Vidobeobachtung<br>(modifizierte Basstechnik, OFF-Modus)<br>Plaqueprobe, Plaqueanfärbung, Plaqueindizes, Foto	
	1 Woche ↓	keine Interdentalraumhygiene
5. Termin	4 Tage vorher: keine Mundhygienemassnahmen<br>Zahnputzinstruktion (Schallzahnbürste)<br>Plaqueprobe, Plaqueanfärbung, Plaqueindizes, Foto<br>Zähneputzen unter Vidobeobachtung<br>(Putztechnik, ON-Modus)<br>Plaqueprobe, Plaqueanfärbung, Plaqueindizes, Foto<br>Professionelle Zahnreinigung	

5.4 Mikrobiologische Verfahren

Der Interdentalraum wird mit Luft getrocknet und mit Hilfe von Sauger und Watterollen trocken gehalten. Die supragingivale Plaque wird mit sterilen Küretten aus dem Interdentalraum entnommen und separat in ein steriles, mit Transportmedium (Reduced Transport Fluid, RTF) [Syed and Loesche, 1972] gefülltes Röhrchen gegeben. Die Proben werden während einer Minute gevortext, Glycerol zu einer Endkonzentration von 20% beigegeben und anschliessend bei -80°C gelagert.

Die Gesamtkeimzahl sowie die Anzahl ausgewählter oraler Bakterien wird sowohl durch Kultur wie auch über eine quantitative real-time PCR bestimmt. Zur Ermittlung der Gesamtlebendzahl (CFU/ml) sowie des Anteils schwarz pigmentierender Kolonien werden die Proben während einer Minute gevortext und entsprechende Verdünnungen auf Blutagarplatten (Columbia Agar Base [BBL Becton Dickinson, Allschwil, Schweiz] ergänzt mit 5mg/L Hemin, 0.5 mg/L Menadion und 50ml/L Blut) ausplattiert, unter aeroben sowie anaeroben Bedingungen bei 37 C° für 3-5 resp. 10 Tage inkubiert und danach ausgezählt. Zur quantitativen Bestimmung mittels real-time PCR wird die genomische DNA der Bakterien aus den Proben isoliert und Teile der 16S rDNA amplifiziert. Dazu werden publizierte Primerpaare sowohl für die quantitative Bestimmung der Gesamtbakterienpopulation (universelles16S rDNA-Primerpaar) als auch für die Amplifikation spezifischer oraler Bakterien verwendet.

Mit Hilfe der Live-Dead-Färbung erfolgt die Bestimmung des Anteils lebender bzw. toter Bakterien. Dazu werden zwei spezifische Fluoreszenzfarbstoffe (Live/Dead BacLight Bacterial Viability Kit; MoBiTec, Luzern, Switzerland) zu den Proben gegeben. Nach entsprechender Inkubationszeit werden die Proben unter einem Fluoreszenzmikroskop (CLSM; Carl Zeiss AG, Oberkochen, Deutschland) analysiert. Die Färbung beruht auf der Durchlässigkeit der Zellmembran. Die toten Zellen fluoreszieren rot, die lebenden Zellen fluoreszieren grün.

5.5 Videobeobachtung und -analyse

Die Videobeobachtung findet in Abwesenheit des Untersuchers über einen Zeitraum von 10 Sekunden statt. Die Probanden verwenden die Schallzahnbürste ohne Zahnpaste. Die Probanden putzen vor einem Spiegel, hinter dem eine Videokamera integriert ist. Das Verfahren ist an anderer Stelle ausführlich beschrieben [Winterfeld, 2015]. Die Videoanalyse erfolgt nach Abschluss des klinischen Teils der Studie mit einer speziellen Software zur Beobachtung von Verhalten (INTERACT® 15, 2015. Mangold International GmbH, Arnstorf, Deutschland). Das Verfahren ist an anderer Stelle ausführlich beschrieben [Winterfeld, 2015].

Folgende Parameter werden erhoben:

- Bürstbewegung (kreisend, horizontal-linear, vertikal linear, vertikal-rollend, rüttelnd, rüttelnd-auswischend (modif. Bass), passiv = Bürstkopf wird nur am Zahn positioniert, keine eigenen Bürstbewegungen).

5.6 Planimetrische Bestimmung der Plaquemenge

Die Bukkalflächen der beiden Prämolaren und des ersten Molaren werden formatfüllend digital fotografiert. Angefärbte Plaque wird computergestützt planimetrisch quantifiziert.

Dazu werden die Farbfotos zunächst in Graustufen umgewandelt (Adobe Photoshop Lightroom 5.4; Adobe Systems; San José, California, USA). Auf einer 8-bit Graustufenskala entspricht 0 reinem Schwarz und 255 reinem Weiß. Der Grenzwert für die Definition eines Pixels als „plaquebedeckt“ wird bei 90 festgelegt. Die weitere Verarbeitung (Auswahlmaske und automatische Quantifizierung) erfolgt mit ImageJ version 1.47q (Wayne Rasband; National Institute of Mental Health, Bethesda, Maryland, USA). Die Plaquemenge wird als prozentualer Anteil der plaquebedeckten Zahnfläche an der Gesamtzahnfläche (P%) beschrieben.
Zielzähne sind die beiden Prämolaren und der erste Molar.

5.7 Randomisierung und Verblindung

Vor Beginn der Studie werden zwei computergenerierte Randomisierungslisten (Entnahmeort der Plaqueproben vor und nach dem Putzen; Microsoft Office Excel® 2011, Microsoft Corp., Redmond, WA, USA) sowie eine geeignete Liste mit Probandencodes erstellt. Die Intervention ist für alle Probanden identisch, sodass sich die Randomisierung lediglich auf den Ort der Plaqueentnahme bezieht.
Die Auswertung der Videos (Freiburg) erfolgt in Bezug auf das Wissen um die Intervention verblindet, allerdings kann ein Erkennen der Intervention aufgrund der veränderten Putzgewohnheiten nicht vollständig ausgeschlossen werden. Die mikrobiologischen Analysen (Basel) sowie die planimetrische Plaquebestimmung (Gießen) erfolgen gegenüber der Intervention verblindet.

5.8 Prüfung der Studie durch Ethik-Kommisionen

Vor Studienbeginn wird das Vorhaben zunächst der Ethik-Kommission der Universität Freiburg vorgelegt, das Vorhaben wird anschließend mit positivem Votum der Ethik-Kommission Freiburg den Ethik-Kommissionen der Universitäten Basel und Gießen zur Kenntnis gegeben sowie ggf. deren positive Voten abgewartet. Die Studie wird im Register für klinische Studien registriert.

6 Plan zur statistischen Analyse und Fallzahlberechnung

Die statistische Auswertung erfolgt mit SPSS (SPSS® Statistics 23; SPSS Inc., Chicago, IL, USA). Die statistische Analyse erfolgt in Basel.

Verhältnisskalierte Daten (P%, Gesamtkeimzahl, Anteil schwarzpigmentierender Kolonien, Anteil lebender bzw. toter Bakterien):
Die Daten werden auf signifikante Abweichungen von der Normal-Verteilung geprüft.
Ob zwischen der planimetrisch bestimmten Plaquemenge auf der Zahnoberfläche (P%) und der mikrobiologisch bestimmten Plaquemenge im Interdentalraum nach Putzen im ON gegenüber dem OFF Modus unter den verschiedenen Putztechniken (Bass-Technik im OFF Modus versus habituelle Technik im OFF Modus; Bass-Technik im OFF Modus versus korrekte Technik im ON Modus; Habituelle Technik im OFF versus habituelle Technik im ON Modus; Instruierte Anwendung im ON Modus versus habituelle Anwendung im ON Modus) Unterschiede bestehen, wird mit t-Tests für abhängige Stichproben untersucht.

Gegebenfalls kommen parameterfreie Verfahren zur Anwendung.
Ordinalskalierte Daten (klinische Plaquescores):
Ob zwischen den Plaquescores nach Putzen im ON gegenüber dem OFF Modus unter den verschiedenen Putztechniken Unterschiede bestehen, wird mit dem Wilcoxon-Test untersucht.

Die Verfahren für die statistische Auswertung werden nach Abschluss der Datenerhebung finalisiert.

Fallzahlberechnung:
Die Fallzahlberechnung basiert auf einer Studie, in die eine vergleichbare Studienpopulation eingeschlossen wurde. In dieser wurden Plaquewerte nach einer Zahnputzabstinenz für etwa 12h (Auslassen der Mundhygiene am Abend zuvor) ermittelt. Es konnte ein mittlerer Plaquewert von 2,0±0,5 gemessen werden. Die anschließend durchgeführte Mundhygiene mit einer manuellen Zahnbürste erzielte eine Plaquereduktion auf einen mittleren Plaquewert von 1,2±0,5 [Van der Weijden et al., 2002].
Die Hälfte der dort gemessenen Reduktion kann als klinisch relevant gewertet werden (Plaquewert-Reduktion um 0,4). Unter der Annahme von $\alpha = 0{,}05$ und $ß = 0{,}2$ sowie einer Standardabweichung von 0,5 ergibt sich eine Gruppengröße von 23. Unter der Berücksichtigung von eventuellen Ausfällen wird daher eine Gruppengröße von 30 geplant [Schlueter et al., 2010].

7 Kalibrierung und Training

Die Studienverantwortlichen stellen sicher, dass die an der klinischen Studie ebenso wie die an den mikrobiologischen und plaqueplanimetrischen Auswertungen beteiligten Personen ausführlich über das Vorhaben und die Zielsetzung sowie den Ablauf der Studie informiert sind.
Der klinische Untersucher (Freiburg) wird anhand von Fotos mit angefärbter Plaque in Bezug auf die klinischen Plaqueindizes kalibriert. Zusätzlich lernt der klinische Untersucher anhand von bereits bestehenden Videos diese nach oben genannten Kriterien reproduzierbar auszuwerten.
Die planimetrische Bestimmung der Plaquemenge (Gießen) erfolgt computergestützt automatisiert durch einen erfahrenen Untersucher, Daten zur Reproduzierbarkeit werden jedoch auch für diesen Teil der Studie generiert.

8 Dokumentation

Alle handschriftlichen Daten werden auf Formblättern dokumentiert (Case Report Form, CRF), die nur den Probandencode enthalten. Die Eintragungen erfolgen leserlich und vollständig. Die Film- und Fotodateien werden nur unter dem Probandencode abgespeichert. Von den Dateien werden Sicherheitskopien angefertigt.
Der „Informed Consent“ ist das einzige Dokument, das die Zuordnung von Proband und Probandencode erlaubt und wird getrennt von allen anderen Dokumenten aufbewahrt.

Die handschriftlichen Daten und die mit INTERACT gewonnenen Daten werden in SPSS überführt, auf Korrektheit (handschriftliche Daten) bzw. auf Plausibilität (INTERACT Daten) geprüft und ausgewertet.
Die Archivierung der Studiendokumente einschließlich aller Rohdaten erfolgt in Freiburg.

9 Audits und Kontrollen zu Qualitätssicherung

Die Studienverantwortlichen stellen an ihren Zentren den protokollgemäßen Ablauf der Studie sicher und gewährleisten die Vollständigkeit der Dokumentation sowie die Qualität der Daten. Alle Zentren ermöglichen sich den vollständigen Zugang zum Studienmaterial und den Probandendaten. Dabei wird die Anonymität des Probanden gewahrt, die Daten werden vertraulich gehandhabt.

10 Protokolländerungen

Änderungen oder Abweichungen vom Protokoll werden nur nach Diskussion mit allen an der Studie involvierten Personen zugelassen. Die zuständige(n) Ethikkommission(en) wird/werden von Protokolländerungen in Kenntnis gesetzt und muss/müssen zustimmen, wenn es sich um wesentliche Änderungen handelt (bspw. Erhebung zusätzlicher Daten).

11 Publikation

Die Ergebnisse der Studie sollen möglichst hochrangig publiziert werden. Die Ergebnisse der quantitativen/qualitativen mikrobiologischen Plaqueanalyse der klinischen Studie werden unter Erst- (Position A) und Letztautorschaft der Gruppe Basel mit geteilter Erstautorschaft Freiburg/Gießen (Position B), die Ergebnisse der quantitativen klinischen Plaqueanalyse der klinischen Studie unter der Erst- (Position A) und Letztautorschaft der Gruppe Freiburg/Gießen mit geteilter Erstautorschaft (Position B) Basel publiziert.
Die Ergebnisse der quantitativen klinischen Plaqueanalyse der klinischen Studie (Freiburg) sind außerdem Gegenstand der Dissertation von N.N.

12 Literatur

Lange DE, Plagmann HC, Eenboom A, Promesberger A: Clinical methods for the objective evaluation of oral hygiene. Dtsch Zahnarztl Z 1977;32:44-47.

Rustogi KN, Curtis JP, Volpe AR, Kemp JH, McCool JJ, Korn LR: Refinement of the Modified Navy Plaque Index to increase plaque scoring efficiency in gumline and interproximal tooth areas. J Clin Dent 1992;3 (Suppl C):C9-C12.

Saxer UP, Imfeld T, van Waes H: Medienmitteilung "Hydrodynamik-Schallzahnbürsten", SSO Taskforce 2010. Schweiz Monatsschr Zahnheilkd 2005.

Schlueter N, Klimek J, Saleschke G, Ganss C: Adoption of a toothbrushing technique: a controlled, randomised clinical trial. Clin Oral Invest 2010;14:99–106.

Schmidt JC, Astasov-Frauenhoffer M, Hauser-Gerspach I, Schmidt JP, Waltimo T, Weiger R, Walter C: Efficacy of various side-to-side toothbrushes for noncontact biofilm removal. Clin Oral Investig 2014;18:793-800.

Schmidt JC, Astasov-Frauenhoffer M, Waltimo T, Weiger R, Walther W: Efficacy of various side-to-side toothbrushes and impact of brushing parameters on noncontact biofilm

removal in an interdental space model. Clin Oral Investig 2016;Oct 19. [Epub ahead of print].

Schmidt JC, Zaugg C, Weiger R, Walter C: Brushing without brushing?--a review of the efficacy of powered toothbrushes in noncontact biofilm removal. Clin Oral Investig 2013;17:687-709.

Sharma PK, Gibcus MJ, van der Mei HC, Busscher HJ: Influence of fluid shear and microbubbles on bacterial detachment from a surface. Appl Environ Microbiol 2005;71:3668-3673.

Syed SA, Loesche WJ: Survival of human dental plaque flora in various transport media. Appl Microbiol 1972;24:638-644.

Van der Weijden GA, Timmerman MF, Piscaer M, Snoek I, Van der Velden U, Galgut PN: Effectiveness of an electrically active brush in the removal of overnight plaque and treatment of gingivitis. J Clin Periodontol 2002 Aug 1;29:699–704.

Weiger R, Netuschil L, Brecx M: Relationship between bacterial counts, microbial vitality and the accumulation of supragingival dental plaque in humans. J Periodontal Res 1992;27:575-580.

Winterfeld, T. Toothbrushing and flossing habits in young adults: a video-based observational study. Med Diss, Gießen, 2015.
